# The anabolic steroid stanozolol is a potent inhibitor of human MutT homolog 1

**DOI:** 10.1002/1873-3468.70116

**Published:** 2025-07-27

**Authors:** Emma Scaletti Hutchinson, Robert Gustafsson Westergren, Ingrid Almlöf, Ann‐Sofie Jemth, Martin Scobie, Ulrika Warpman Berglund, Thomas Helleday, Pål Stenmark

**Affiliations:** ^1^ Department of Biochemistry and Biophysics Stockholm University Stockholm Sweden; ^2^ Department of Oncology‐Pathology, Science for Life Laboratory Karolinska Institutet Stockholm Sweden

**Keywords:** anabolic steroid, enzyme inhibition, MTH1, Nudix enzyme, stanozolol

## Abstract

Human MutT homolog 1 (hMTH1) removes damaged nucleotides from the nucleotide pool, preventing their incorporation into DNA. Due to its potential as an anticancer drug target, hMTH1 has been the focus of several inhibitor development studies. Unexpectedly, we show that the anabolic steroid stanozolol (Stz) is a potent nanomolar inhibitor of hMTH1. We present the structure of hMTH1 in complex with Stz, which indicates a unique core scaffold that could be exploited for future inhibitor development. Comparison with human protein structures bound with dihydrotestosterone (DHT) shows hMTH1 is entirely unrelated in terms of its structure. As these DHT binding proteins are all involved in steroid regulation, this makes the identification of Stz as a potent hMTH1 inhibitor all the more unusual.

## Graphical abstract

MutT homolog 1 (MTH1) is a member of the NUDIX superfamily of enzymes and is an anticancer drug target. We show that stanozolol (Stz), an anabolic steroid, is an unexpected nanomolar inhibitor of MTH1. The X‐ray crystal structure of the human MTH1–Stz complex reveals a unique binding scaffold that could be utilized for future inhibitor development.

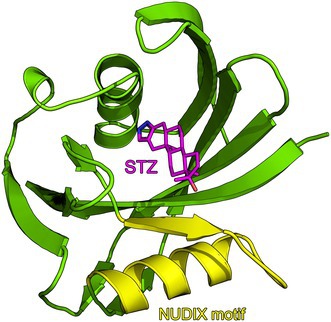

## Abbreviations

**17β‐HSD1**, 17‐β‐hydroxysteroid dehydrogenase type 1

**AR**, androgen receptor

**DHT**, dihydrotestosterone

**hMTH1**, human MutT homolog 1

**LBD**, ligand binding domain

**Nudix**, nucleoside diphosphate linked to some moiety x

**PDB**, Protein Data Bank

**ROS**, reactive oxygen species

**SHBG**, Sex hormone‐binding globulin

**Stz**, stanozolol

Human MutT homolog 1 (hMTH1) belongs to the Nudix (Nucleoside diphosphate linked to some moiety x) protein superfamily, which is characterized by the conserved amino acid motif GX_5_EX_7_REUXEEXGU, where U is a hydrophobic residue [1]. MTH1 was first identified to hydrolyze oxidized dNTPs such as 8‐oxo‐dGTP and 2‐oxo‐dATP [2, 3]; however, in recent years, methylated dNTPs such as O6‐methyl‐dGTP and N6‐methyl‐dATP have also been identified as natural substrates of the enzyme [4, 5]. MTH1 sanitizes the free nucleotide pool by hydrolyzing these non‐canonical dNTPs into their monophosphate forms, thereby preventing their incorporation into DNA, and thus reducing genotoxicity [4, 6]. MTH1 is overexpressed in many cancers and is essential for the survival of several cancer cell lines [7]; however, the enzyme is dispensable for the growth of untransformed cells [8, 9]. This is proposed to be a result of the significantly higher levels of reactive oxygen species (ROS) in cancer cells compared to healthy cells, which damage the free nucleotide pool. Inhibition of MTH1 can be fatal for cancer cells because they replicate so rapidly and often lack the control mechanisms present in normal cells that prevent DNA damage [10]. Furthermore, it was recently shown that MTH1 inhibition results in synthetic lethality in MYC over‐expressing cells, which decreases tumorigenic potential *in vivo* [11]. This is important as, at present, MYC family oncoproteins are largely considered to be undruggable [12]. Due to its promise as an anticancer drug target, numerous small molecule MTH1 inhibitors have been developed [8, 13, 14, 15, 16, 17, 18, 19]. Interestingly, not all potent MTH1 inhibitors kill cancer cells, and the validity of MTH1 as a cancer target has therefore been questioned [20, 21, 22, 23]. However, it has been suggested that these inhibitors fail to enhance oxidative damage in DNA, which may be why these compounds do not kill cancer cells [9]. It is evident that MTH1 biology is more complex than previously thought, and recent studies have shown that, in addition to targeting MTH1, certain inhibitors also arrest cells in mitosis through inhibition of tubulin polymerization, which is likely both dependent and independent of MTH1, resulting in oxidative damage to DNA, which kills cancer cells [9, 24].

**Fig. 1:**
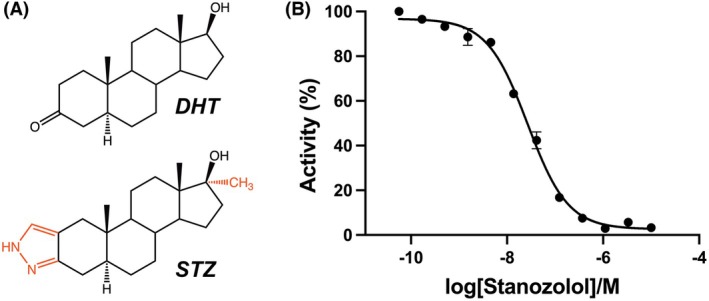
(A) Chemical structures of Dihydrotestosterone (DHT) and Stanozolol (STZ). Key structural differences are shown in red. (B) Stanozolol dose–response curve for human MTH1. IC50 = 26 nm (*n* = 3). Data are shown as mean ± standard deviation (SD). In this experiment, dGTP was used as the assay substrate. Refer to the Materials and Methods section for details on experiment procedures.

As there are many described inhibitors of MTH1, we decided to screen an in‐house library containing commercially available drug compounds, and unexpectedly identified the steroid stanozolol (Stz) as a potent inhibitor of hMTH1. Stz is a synthetic steroid derived from dihydrotestosterone (DHT) (Fig. 1A), which is marketed under the names Stanozolol®, Stromba®, and Winstrol® [25]. Anabolic steroids such as Stz greatly increase the size and strength of skeletal muscle by activating the androgen receptor (AR), a DNA‐binding transcription factor that regulates the expression of genes specific to muscle development [26, 27]. Anabolic androgenic steroids (AAS) were originally developed for medical purposes, such as treating hormone deficiencies and muscle‐wasting diseases [28]. However, due to their positive effects on muscle mass, strength, and endurance, AAS are also used at supra‐physiological levels by athletes, which can greatly improve their athletic abilities [29, 30]. Due to their unfair performance‐enhancing qualities, Stz and other AAS are classified as banned substances by the World Anti‐Doping Agency [31] and were first banned by the International Olympic Committee in 1974 after methods to detect them had been developed [32]. Arguably, one of the most well‐known cases of Stz abuse in professional sport occurred at the 1988 Seoul Olympic Games when Canadian Ben Johnson was stripped of his gold medal for the 100 metre sprint after testing positive for the steroid [33]. In addition to its misuse in sport, Stz use is also prevalent amongst professional and amateur bodybuilders [34, 35]. The chemical structures of AAS have been modified to promote their anabolic (muscle building) effects while minimizing their androgenic (masculinizing) qualities. However, while the androgenic effects with AAS have been reduced, they have not been eliminated, which can cause severe health problems when they are used at supra‐physiological concentrations [36]. AAS misuse disrupts the body's natural hormone balance and can lead to aggression, cardiovascular issues (cardiomyopathy, myocardial infarction, fatal arrhythmias), liver damage (cholestasis, hepatic necrosis), and reproductive system abnormalities (hypogonadism, infertility) [28, 37, 38, 39, 40]. There have even been reports where otherwise healthy amateur bodybuilders have died due to Stz‐related complications [41, 42].

Here we report the structure of hMTH1 in complex with Stz, which is the first structure of any protein in complex with this anabolic steroid. We show that Stz is a potent nanomolar inhibitor of hMTH1, and its core structure represents a novel scaffold that could be utilized for future inhibitor development. Due to its important role in sanitizing the free nucleotide pool of oxidatively damaged dNTPs, hMTH1 inhibition by Stz may potentially exacerbate the effects of oxidative damage observed with Stz usage. Further studies are needed to evaluate the role of hMTH1 inhibition in the process of oxidative damage during prolonged use of anabolic steroids.

## Materials and methods

### Protein expression and purification

*H. sapiens mth1* cDNA optimized for *E. coli* expression was purchased from Geneart AG and subcloned into a pET28a(+) expression vector (Novagen, Darmstadt, Germany). hMTH1 was expressed in *E. coli* BL21 (DE3) cells at 18 °C overnight in Terrific Broth media (Formedium) following induction with 0.5 mm IPTG. The cells were harvested, resuspended, and lysed via sonication. His‐tagged hMTH1 was purified using a 5 mL His‐Trap HP (GE Healthcare, Amersham, UK) after which the His‐tag was removed by incubation overnight with thrombin protease (Novagen). The sample was then loaded onto a 5 mL His‐Trap HP column, after which the flow‐through fractions containing tag‐free hMTH1 were further purified using a Superdex200 16/600 size‐exclusion column (GE Healthcare). Buffers used for protein purification are listed in Table S1. The protein was verified to be >95% pure by SDS/PAGE. Purified hMTH1 was aliquoted and stored at −80 °C.

### MTH1 enzyme inhibition assay

To assess the extent of hMTH1 inhibition in the presence of Stz or DHT, hMTH1 activity was determined at a range of compound concentrations using a modified Malachite Green assay [43]. Stz and DHT were serially diluted in DMSO in a 1:3 dilution series and then further diluted in assay buffer (100 mm Tris‐acetate, 40 mm NaCl, 10 mm Magnesium acetate, 1 mm DTT, and 0.005% Tween20) resulting in 12 different Stz and DHT concentrations ranging from 10 mm to 53.3 pm. The final concentration of DMSO in each assay well was 1%. Purified hMTH1 was added to a final concentration of 6 nm. dGTP was added to a final concentration of 100 μm, and the final concentration of inorganic pyrophosphatase was 0.2 U·mL^−1^. When determining the IC50 of Stz in the presence of 8‐oxo‐dGTP, the nucleotide was added to a final concentration of 15 μm and the final hMTH1 concentration was 1.25 nm. In all cases, the final assay volume was 100 μL. Samples lacking enzyme (negative control) or inhibitor (positive control) were included on the assay plate. The reaction mixture was incubated for 15 min at 22 °C, after which 25 μL malachite green assay reagent was added, and the assay plate was further incubated for 15 min before absorbance at 630 nm was read. Assay points were run in triplicate. IC50 values were determined using nonlinear regression by fitting the curve log [inhibitor] vs. response to the data using GraphPad Prism 6.0 software (La Jolla, California, USA). Stz and DHT were purchased from Sigma‐Aldrich, and nucleotide substrates were purchased from (Jena Biosciences, Jena, Germany).

### Crystallization

Purified hMTH1 (11 mg·mL^−1^) was preincubated with 5 mm Stz for 1 h on ice. The protein was crystallized via sitting drop vapor diffusion with 0.1 m sodium acetate pH 4.0, 0.2 m lithium sulfate, 25% (w/v) PEG6000 at 22 °C using a 1:1 and 1:2 precipitant to protein ratio. hMTH1 crystals, which appeared within 5 days, were cryo‐protected by soaking in mother liquor supplemented with 20% (v/v) glycerol and were subsequently flash‐frozen in liquid nitrogen.

### Data collection, structure solution, and refinement

X‐ray diffraction data were collected at beamline 14.1 of the BESSY Light Source (Berlin, Germany). A complete dataset was collected on a single crystal at 100 K. The data were processed and scaled with XDS [44] and Aimless [45] within the CCP4 suite [46]. Molecular replacement was performed in Phaser [47] using the structure of human MTH1 (PDB ID: 3ZR1) with ligands and waters removed, as the search model. Several cycles of model building and refinement were performed using Coot [48] and Refmac5 [49] during which waters and ligands were incorporated into the structure. Data processing and refinement statistics are presented in Table 1. The coordinates and structure factors for hMTH1‐Stz were deposited in the PDB under the accession code 9GQL.

**Table 1:** Data collection and refinement statistics for hMTH1‐Stz complex. Values in parentheses are for the highest resolution shell.

**Data collection**	
Space group	P22_1_2_1_
Cell dimensions	
a, b, c (Å)	36.5, 59.7, 66.9
No. observations	209 462 (7695)
No. unique reflections	29 373 (1322)
Resolution (Å)	44.5–1.40 (1.42–1.40)
*R*_merge_	0.048 (0.525)
*R*_pim_	0.028 (0.339)
CC(1/2)	1.000 (0.666)
I/σI	18.9 (2.7)
Completeness	99.5 (92.3)
Redundancy	7.1 (5.8)
**Refinement**	
*R*_work_/*R*_free_ (%)	14.8/19.2
*B*‐factors	
Wilson *B*‐factor	11.6
Protein	18.0
Ligand	31.7
Water	38.5
R.m.s. deviations	
Bond lengths (Å)	0.013
Bond angles (°)	2.06
Ramachandran plot (%)	
Favored	100
Disallowed	0
RSCC Stz	0.94

## Results and discussion

### Inhibition of hMTH1 by Stz

Following expression in *E. coli*, hMTH1 was purified to homogeneity by Immobilized metal affinity (IMAC) and Size‐exclusion (SEC) chromatography (refer to Materials and Methods section). We produced Stz and DHT dose–response curves for purified hMTH1, which indicated an IC50 value of 26 nm for Stz (Fig. 1B) while DHT did not show any inhibition of hMTH1 (Fig. S1) showing the importance of the methyl group and/or the pyrazole ring for hMTH1 inhibition. Although dGTP is not the preferred substrate of hMTH1, we used it in our assays because it allows testing at concentrations below its Km value. To confirm the relevance of our findings, we also determined the IC50 value of Stz using the preferred hMTH1 substrate, 8‐oxo‐dGTP, which binds in the same position in the enzyme's active site as dGTP (Fig. S2). We performed the assay with 15 μm 8‐oxo‐dGTP, a concentration close to its Km value of approximately 12.5 μm, as lower concentrations are challenging to use with the current assay system. The resulting IC50 of 30 nm was comparable to that obtained with dGTP, supporting the use of dGTP as a suitable substrate in these assays. Due to its potential as an anticancer drug target, hMTH1 has been the focus of several inhibitor development campaigns. In the Protein Data Bank (PDB), there are 69 individual hMTH1 structures in complex with inhibitors, of which 55 represent unique inhibitor molecules (Table S2). In regards to hMTH1 inhibition, Stz ranks highly amongst existing inhibitors, with only 15 of the aforementioned compounds being equally, or even more potent than the steroid [8, 15, 18, 20, 21, 22, 50]. We therefore aimed to determine the crystal structure of hMTH1 in complex with Stz to assess the similarities and differences in binding compared to previously solved hMTH1 inhibitor‐bound structures.

### Overall structure of hMTH1‐stanozolol complex

**Fig. 2:**
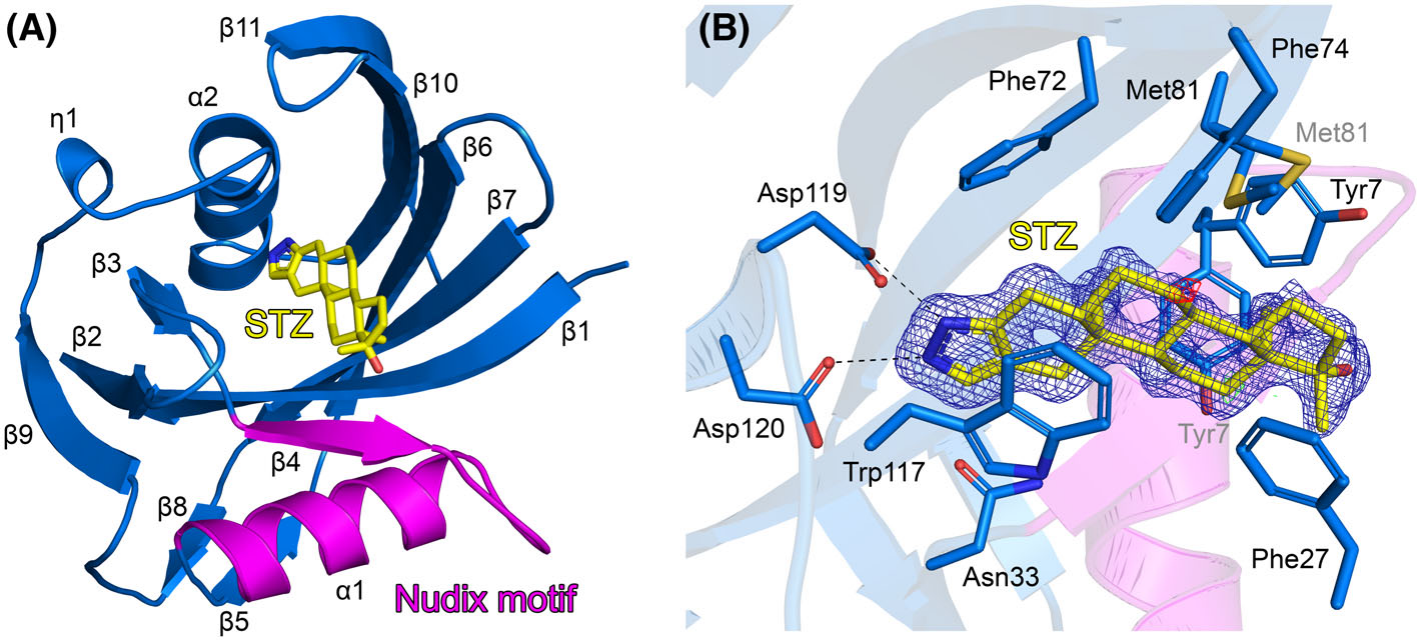
Crystal structure of hMTH1‐stanozolol complex. (A) Ribbon representation of the hMTH1 monomer, colored blue. The highly conserved Nudix motif is colored magenta. The stanozolol (STZ) ligand is depicted as a stick model; C atoms colored yellow, O atoms red, and N atoms dark blue. The secondary structure elements, alpha‐helices (α1‐2), beta‐strands (β1‐11) and 3_10_‐helices (η1) are labeled. (B) The active site hydrogen bond network of hMTH1 with stanozolol. Amino acids contributing to ligand binding are shown as sticks; C atoms are colored blue, O atoms red, N atoms dark blue, and S atoms gold. Hydrogen bond interactions are shown as dashed lines. The alternative conformations of Tyr7 and Met81 are labeled in gray. The 2*F*o‐*F*c electron density map around STZ is contoured at 1.5 σ (blue), and the *F*o‐*F*c electron density maps are contoured at −3.0 σ (red) and + 3.0 σ (green). Figure produced with PyMOL (version 3.0.4, Schrödinger).

The X‐ray crystal structure of hMTH1 bound to Stz (hMTH1‐Stz) was solved to 1.40 Å resolution. The structure of hMTH1 is a monomer (Fig. 2A), consistent with previous studies of hMTH1 which have shown the protein to be monomeric in solution [2, 4, 8]. The hMTH1 monomer is composed of two α‐helices (α1 and α2), eleven β‐strands (β1 to β11), and one 3_10_‐helix (η1) and has an α/β/α sandwich fold which is conserved amongst members of the Nudix protein superfamily [51, 52, 53, 54]. The highly conserved Nudix motif (GX_5_EX_7_REUXEEXGU) is located on α‐helix 1 and β‐strand 4 of hMTH1 (Fig. 2A). This important motif contains the residues required for metal binding and hydrolysis of nucleotide triphosphate substrates [2, 5, 55]. In our hMTH1 structure, no metal ions are present as they are not required for the binding of Stz. Cα‐atom superposition of hMTH1‐Stz with apo hMTH1 (PDB ID: 3ZR1) shows that no major structural rearrangements occur upon Stz binding, as indicated by the low RMSD value of 0.73 Å. However, there are slight differences in the positions of the loop connecting β‐strands 2 and 3 close to the substrate binding site, as well as the loop connecting β‐strands 8 and 9, which is distant to the active site (Fig. S3A).

### Stanozolol recognition by hMTH1

The hMTH1 structure showed unambiguous electron density for Stz, which was able to be placed in the model at full occupancy. The steroid is positioned by hydrogen bonding with Asp119 and Asp120 and an important π‐stacking interaction with the active site tryptophan Trp117. In addition, Stz is further supported by extensive van der Waals interactions with residues Tyr7, Phe27, Asn33, Phe72, Phe74, and Met81 (Fig. 2B). Alternative conformations were observed for both Tyr7 and Met81, which position the end of Stz that is closest to the surface of the protein. Comparison with apo hMTH1 shows that amino acids involved in the binding of Stz generally superimpose very well between the structures (Fig. S3B). The exception to this is Phe27, which shifts significantly due to the movement of the loop connecting β‐strands 2 and 3 (Fig. S3A). Interestingly, no alternative conformations were observed for any residues in the binding pocket of the apo structure. However, this could be a result of our 1.40 Å hMTH1‐Stz structure being significantly higher resolution than the 1.90 Å hMTH1 apo structure.

### Comparison of hMTH1‐Stz with existing structures of human proteins bound to DHT

**Fig. 3:**
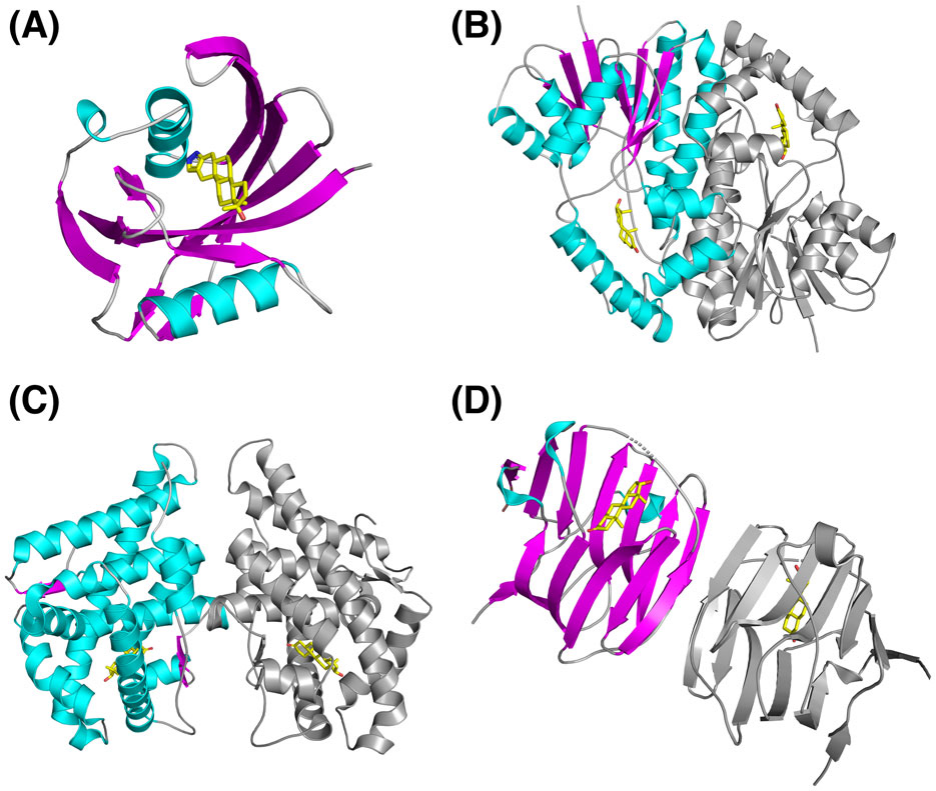
Comparison of human MTH1‐stanozolol complex and other DHT‐bound human proteins. X‐ray crystal structures of (A) hMTH1‐Stz, (B) 17‐β‐hydroxysteroid dehydrogenase type 1 (17β‐HSD1, PDB ID: 1DHT), (C) Androgen receptor (AR) ligand binding domain (PDB ID: 5JJM) and (D) Sex hormone‐binding globulin (SHBG, PDB ID: 1D2S) visualized as ribbon representations; α‐helices colored magenta, β‐strands colored cyan, and loops colored light gray. For homodimeric structures, the second monomer is colored gray. The ligands Stz (panel A) and DHT (panels B–D) are shown as sticks; C atoms colored yellow, O atoms red, and N atoms dark blue. Figure produced with PyMOL (version 3.0.4, Schrödinger).

As our hMTH1‐Stz complex is the only available structure of a protein bound to Stz, we searched the PDB for human proteins bound to dihydrotestosterone (DHT), the steroid from which Stz is derived, which is the most structurally similar compound to Stz in the PDB. Although DHT does not inhibit hMTH1, we aimed to explore whether proteins that bind DHT share any structural similarities with hMTH1 due to the shared steroidal core of DHT and Stz (Fig. 1A). This analysis revealed that there are 43 individual X‐ray crystal structures of *H. sapiens* proteins bound to DHT. This corresponds to three different proteins: (1) 17‐β‐hydroxysteroid dehydrogenase type 1 (17β‐HSD1) [56, 57], (2) Sex hormone‐binding globulin (SHBG) [58, 59, 60], and (3) Androgen receptor ligand binding domain (AR‐LBD) [61, 62, 63, 64, 65, 66, 67, 68, 69] (Table S3). Evidently, the vast majority of the DHT‐bound structures in the PDB are of the *H. sapiens* AR‐LBD. Comparison with these proteins indicates that hMTH1 is entirely unrelated in terms of its amino acid sequence identity and tertiary structure. Furthermore, hMTH1 is exclusively monomeric, whereas 17β‐HSD1, SHBG, and the AR‐LBD exist as homodimers (Fig. 3). However, it should be noted that dimerization of the AR‐LBD is only induced by agonists of the receptor [67]. Unsurprisingly, 17β‐HSD1, SHBG, and the AR‐LBD are all important for the regulation of steroid hormones. For example, 17β‐HSD1 is involved in estrogen activation and androgen inactivation [70], SHBG binds androgens and estrogens, which influences the local bioavailability of sex hormones in the blood and determines which tissues these hormones can access [71], and the AR‐LBD is a nuclear transcription factor that mediates the action of androgens in the body [72]. This contrasts markedly with human MTH1, which primarily sanitizes the free nucleotide pool from damaged nucleotide triphosphates [4, 73] and has no known role in the regulation of steroids.

### Comparison of hMTH1‐Stz with existing hMTH1 nucleotide‐bound structures

**Fig. 4:**
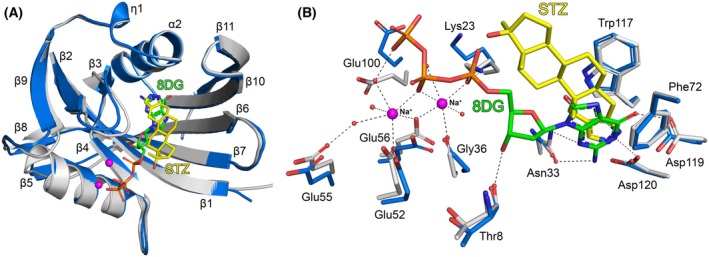
Comparison of hMTH1‐Stanozolol and hMTH1‐8‐oxo‐dGTP structures. (A) Cα‐atom superposition of whole monomers visualized as ribbon representations. The hMTH1‐Stz structure is colored blue, and hMTH1‐8‐oxo‐dGTP (PDB ID: 5GHI) is colored light gray. The ligands are depicted as a stick models; C atoms colored yellow (Stz) or green (8DG: 8‐oxo‐dGTP), O atoms red, and N atoms dark blue. Sodium ions from the 8‐oxo‐dGTP bound structure are shown as magenta spheres. The secondary structure elements, α‐helices (α1‐2), β‐strands (β1‐11) and 3_10_‐helices (η1) are labeled. (B) Comparison of the substrate binding pockets of hMTH1‐Stz and hMTH1‐8‐oxo‐dGTP. 8‐oxo‐dGTP (8DG) and Stz are depicted in an identical manner to panel A. Water molecules are shown as small red spheres. Interactions between 8‐oxo‐dGTP, sodium ions, and hMTH1 are shown as dashed lines. Figure produced with PyMOL (version 3.0.4, Schrödinger).

The substrate specificity of hMTH1 has been extensively studied, which has shown the enzyme is most active towards oxidized deoxynucleotide‐triphosphate substrates such as 8‐oxo‐dGTP [2, 3, 74, 75, 76] and methylated dNTPs including O6‐methyl‐dGTP [4] and N6‐methyl‐dATP [5]. In the PDB, there are a total of 24 hMTH1 structures in complex with either nucleotide triphosphate substrates or nucleotide monophosphate products, which correspond to 7 unique ligands (Table S4). Comparison of our hMTH1‐Stz structure with hMTH1 in complex with the products 8‐oxo‐dGMP, O6‐methyl‐dGMP, N6‐methyl‐dAMP, and 2‐oxo‐dAMP [4, 5, 76] and the substrates 8‐oxo‐dGTP, 8‐oxo‐ATP, and 2‐oxo‐dATP [3, 74] (RMSD range = 0.49–0.85 Å) indicates that Stz binds in the same general area of the substrate binding pocket. However, while the nitrogen‐containing ring of Stz is located where the base of the nucleotide substrates/products bind, the rest of the steroid is positioned away from where the ribose and phosphate moieties of those compounds interact (Fig. S4). Superposition of hMTH1‐Stz with hMTH1 in complex with 8‐oxo‐dGTP and two sodium ions which approximate magnesium/manganese binding (PDB ID: 5GHI, [3]) shows that the core protein structures superimpose very well with a low RMSD of 0.79 Å. However, there are two loop regions located between beta‐strands β2‐3 and β8‐9 which are significantly shifted between the structures (Fig. 4A). Detailed comparison of the substrate binding pocket indicates that amino acids Asn33, Phe72, Trp117, Asp119, and Asp120 which position the base of 8‐oxo‐dGTP adopt a similar position in our hMTH1‐Stz structure (Fig. 4B). Each of these residues is also required for optimal positioning of the Stz nitrogen‐containing pyrazole ring, particularly the π‐stacking interaction with Trp117 and the H‐bond interactions with Asp119 and Asp120. These two aspartate residues are known to be important for the broad substrate recognition of hMTH1, as they change protonation state depending on whether an adenine or guanine base is bound [76]. As Stz is the only anabolic steroid containing two nitrogen atoms [77] the same H‐bond interactions with Asp119 and Asp120 would not be possible for DHT or other anabolic steroids. This is supported by the finding that DHT does not inhibit hMTH1 (Fig. S1).

The Nudix motif of hMTH1 (residues 37–59, Fig. 2A) contains residues required for binding divalent metal ions (Mg^2+^ or Mn^2+^), which are essential for efficient substrate hydrolysis [1]. Analysis of substrate binding in the structure of hMTH1 bound with 8‐oxo‐dGTP and two sodium ions shows the metals form coordination bonds with Gly36, Glu52, and Glu56 and four water molecules, two of which are positioned by H‐bonding with Glu55 and Glu100 (Fig. 4B). It should be noted that in this structure, there are sodium ions placed where divalent metal ions are expected to bind, which may be why 8‐oxo‐dGTP was not hydrolyzed during crystallization [3]. Comparison with hMTH1‐Stz (RMSD = 0.79 Å) shows that in terms of important residues from the Nudix motif, Glu55 occupies the same position, whereas Glu52 and Glu56 are shifted relative to hMTH1‐8‐oxo‐dGTP. Furthermore, residues Lys23 and Glu100 adopt significantly different positions in the structures. These two amino acids are located in the loop regions between beta‐strands β2‐3 and β8‐9, which represent the areas of secondary structure that differ most between hMTH1‐Stz and hMTH1‐8‐oxo‐dGTP (Fig. 4A). These differences likely result from our hMTH1‐Stz structure being metal free and may therefore represent what occurs in the active site upon binding of divalent metal ions and dNTPs in the native enzyme.

### Comparison of hMTH1‐Stz with existing hMTH1 inhibitor bound structures

**Fig. 5:**
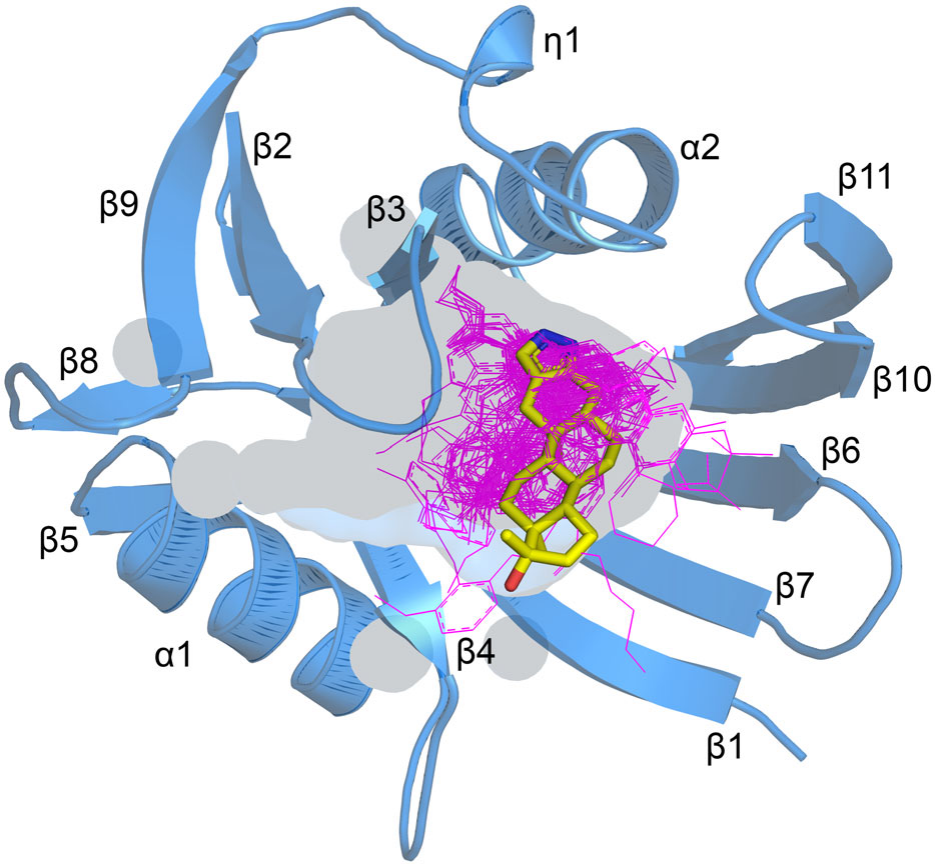
Comparison of hMTH1‐Stanozolol complex with inhibitor bound hMTH1 structures. The Cα‐atoms of hMTH1‐Stz were superimposed with hMTH1 structures bound with various inhibitors (PDB IDs: 3WHW, 4C9X, 4C9W, 4N1U, 4N1T, 5ANW, 5ANV, 5ANU, 5ANT, 5ANS, 5FSN, 5FSM, 5FSL, 5FSO, 5NHY, 5NGT, 5NGS, 5NGR, 6F23, 6F22, 6F20, 6FIX, 6EQ7, 6EQ6, 6EQ5, 6EQ4, 6EQ3, 6EQ2, 6GLV, 6AA5, 6AA4, 6US4, 6US3, 6US2, 6JVT, 6JVS, 6JVR, 6JVQ, 6JVP, 6JVO, 6JVN, 6JVM, 6JVL, 6JVK, 6JVJ, 6JVI, 6JVH, 6JVG, 7N13, 7N03, 8A0T, 8A07, 8A3A, 8A05, 8A34). For clarity, only the monomer of hMTH1‐Stz is shown. The ligand binding cavity of hMTH1‐Stz is shown as a semi‐transparent surface. Stz is depicted as a stick model; C atoms are colored yellow, O atoms are red, and N atoms are dark blue. The inhibitor ligands from the other hMTH1 structures are shown as magenta lines. The secondary structure elements, α‐helices (α1‐2), β‐strands (β1‐11) and 3_10_‐helices (η1) are labeled. Figure produced with PyMOL (version 3.0.4, Schrödinger).

Due to its potential as an anticancer drug target, hMTH1 has been the focus of several inhibitor development campaigns [8, 13, 14, 15, 16, 17, 18, 19, 50]. The PDB contains 69 hMTH1 inhibitor bound structures, of which 55 are unique inhibitors (Table S2). Superposition of hMTH1‐Stz with each of these 55 structures indicates that Stz and the other inhibitors are located in the active site pocket where dNTP substrates bind (Fig. 5). To date, no allosteric inhibitor binding sites on hMTH1 have been identified. hMTH1 is considered to be a highly druggable protein target [8, 14], and visualization of the active site pocket indicates that it is relatively deep and spacious. Analysis of overall protein structures indicates that the core structure of hMTH1‐Stz superimposes very well with the other inhibitor bound structures (RMSD range = 0.36–1.03 Å). However, the loop between beta‐strands β2‐3 in hMTH1‐Stz was shown to be shifted slightly compared to the majority of the hMTH1‐inhibitor structures (Figs S5–S9). In hMTH1‐Stz, residue Phe27 located within this loop forms an important π‐stacking interaction with Stz, which may influence the overall positioning of the loop relative to the other structures. Importantly, while it is evident that all existing hMTH1 inhibitors are located in the same general area of the binding pocket, there are no inhibitors whose core scaffold superimposes with Stz (Fig. 5, Figs S5–S9). Overall, our hMTH1‐Stz structure and analysis indicates that Stz represents a novel scaffold, which could be utilized for future hMTH1 inhibitor development.

## Conclusions

Here, we identified Stz as a potent nanomolar inhibitor of hMTH1 and solved the structure of hMTH1‐Stz; the first structure of any protein in complex with this specific anabolic steroid. This was unexpected as hMTH1 is primarily a nucleotide pool sanitizing enzyme and has not been identified to play any role in the regulation of steroids. Structures of human 17β‐HSD1, SHBG, and the AR‐LBD in complex with DHT have been solved, and as would be expected, each of these proteins is important for regulating steroid hormones in the body. hMTH1 is entirely unrelated to these proteins in terms of its sequence, structure, and function, making its identification as a strong binder of Stz a remarkable finding. hMTH1 has been the focus of several drug development campaigns, and Stz ranks highly among identified inhibitors in terms of its hMTH1 inhibition potency. Importantly, we showed that no existing hMTH1 inhibitors have a core scaffold that superimposes with Stz, and the steroid therefore represents a unique scaffold for future hMTH1 inhibitor development. Further structural alterations/modifications to the core scaffold of Stz may improve its hMTH1 inhibition even further. However, further development of Stz as a specific hMTH1 inhibitor would be highly challenging, as Stz binds other human proteins such as the AR‐LBD; this would need to be minimized while simultaneously improving inhibition toward hMTH1. Our structural data showed Stz is predominantly positioned by hydrophobic interactions due to its large hydrophobic core structure. There were only two H‐bond interactions between Stz and hMTH1, which interestingly involved the two aspartate residues known to be important for the broad substrate specificity of the enzyme. As Stz is the only anabolic steroid containing two nitrogen atoms, similar H‐bond interactions would likely not be possible for DHT or other steroids, which explains the lack of hMTH1 inhibition observed for DHT. As hMTH1 sanitizes the nucleotide pool from oxidatively damaged dNTPs, it is possible that its inhibition by Stz could potentially worsen the effects of ROS induced oxidation. Prolonged use of anabolic steroids at supramolecular levels by athletes and bodybuilders can lead to cardiovascular issues, liver damage, and even death. The exact mechanism of steroid‐induced liver damage is unclear, but proposed mechanisms include cholestasis and, importantly, oxidative stress [28]. A recent study has also shown that misuse of Stz, particularly in combination with cannabis, significantly upregulates oxidative stress damage biomarkers, elevated fibrotic markers, and induces cardiac hypertrophy in rats [78]. This may be particularly relevant for athletes, as in a recent cross‐sectional study 60% of the athletes surveyed admitted to using cannabis at least once, and more than 20% reported that they used cannabis regularly for pain management and recreational purposes [79]. Due to hMTH1 inhibition, nucleotide pool sanitization may be adversely affected in users of anabolic steroids, and athletes are more likely to experience negative effects of exercise‐induced free radical flow, particularly when their workouts are long and intense [80]. Evidently, further studies are required to assess the role and/or extent hMTH1 inhibition plays in the process of oxidative damage during long‐term anabolic steroid use.

## Conflict of interest

Oxcia AB is commercially developing inhibitors of hMTH1, and MS and UWB are employed by Oxcia AB. RGW, IA, MS, UWB, A‐SJ, TH, and PS are shareholders of Oxcia AB. TH is also a member of the Board of Directors of Oxcia AB. The remaining authors declare no competing interests.

## Author contributions

PS and TH conceived the project and supervised the study. UWB was project leader, MS identified Stanozolol as a potent hMTH1 inhibitor. RGW conducted crystallization experiments and determined the structure presented in this paper. ESH performed structure refinement and analyzed data. IA and A‐SJ performed biochemical experiments. ESH wrote the manuscript. All authors provided critical feedback and approved the final version of the manuscript.

## Peer review

The peer review history for this article is available at https://www.webofscience.com/api/gateway/wos/peer‐review/10.1002/1873‐3468.70116.

## Supporting information


**Fig. S1.** Stanozolol and DHT dose–response curves for human MTH1.
**Fig. S2.** Stanozolol dose–response curves for human MTH1.
**Fig. S3.** Comparison of stanozolol‐bound hMTH1 and the hMTH1 apo structure.
**Fig. S4.** Comparison of hMTH1‐Stanozolol complex with hMTH1 nucleotide‐bound structures.
**Fig. S5.** Comparison of stanozolol‐bound hMTH1 and the hMTH1 inhibitor bound structures from Streib *et al*, Huber *et al* and Gad *et al*.
**Fig. S6.** Comparison of stanozolol‐bound hMTH1 and the hMTH1 inhibitor bound structures from Kettle *et al*, Nissink *et al* and Ellermann *et al*.
**Fig. S7.** Comparison of stanozolol‐bound hMTH1 and the hMTH1 inhibitor bound structures from Rudling *et al*, Rahm *et al* and Wiedmer *et al*.
**Fig. S8.** Comparison of stanozolol‐bound hMTH1 and the hMTH1 inhibitor bound structures from Yokoyama *et al*, Farand *et al* and Veits *et al*.
**Fig. S9.** Comparison of stanozolol‐bound hMTH1 and the hMTH1 inhibitor bound structures from Peng *et al*. and unpublished structures.
**Table S1.** hMTH1 protein purification buffers.
**Table S2.** List of inhibitor‐bound human MTH1 structures available in the Protein Data Bank.
**Table S3.** List of human proteins structures in complex with DHT available in the Protein Data Bank.
**Table S4.** List of nucleotide‐bound human MTH1 available in the Protein Data Bank.

## Data Availability

All data can be shared upon request made to the corresponding author. Crystal structure data are deposited in the PDB: 9GQL.
