# Supplementary material for: The anabolic steroid stanozolol is a potent inhibitor of human MutT homolog 1
Source: FEBS Lett. 2025 Jul 27;599(19):2790–801. doi: 10.1002/1873-3468.70116 (PMC12519060; doi:10.1002/1873-3468.70116)
Supplement: Supplementary file 1 — Fig. S1. Stanozolol and DHT dose–response curves for human MTH1. Fig. S2. Stanozolol dose–response curves for human MTH1. Fig. S3. Comparison of stanozolol‐bound hMTH1 and the hMTH1 apo structure. Fig. S4. Comparison of hMTH1‐Stanozolol complex with hMTH1 nucleotide‐bound structures. Fig. S5. Comparison of stanozolol‐bound hMTH1 and the hMTH1 inhibitor bound structures from Streib et al, Huber et al and Gad et al. Fig. S6. Comparison of stanozolol‐bound hMTH1 and the hMTH1 inhibitor bound structures from Kettle et al, Nissink et al and Ellermann et al. Fig. S7. Comparison of stanozolol‐bound hMTH1 and the hMTH1 inhibitor bound structures from Rudling et al, Rahm et al and Wiedmer et al. Fig. S8. Comparison of stanozolol‐bound hMTH1 and the hMTH1 inhibitor bound structures from Yokoyama et al, Farand et al and Veits et al. Fig. S9. Comparison of stanozolol‐bound hMTH1 and the hMTH1 inhibitor bound structures from Peng et al. and unpublished structures. Table S1. hMTH1 protein purification buffers. Table S2. List of inhibitor‐bound human MTH1 structures available in the Protein Data Bank. Table S3. List of human proteins structures in complex with DHT available in the Protein Data Bank. Table S4. List of nucleotide‐bound human MTH1 available in the Protein Data Bank. [file FEB2-599-2790-s001.docx]

**Supporting information**

**Supplementary Table 1. hMTH1 protein purification buffers.**

| Name | Details |
| --- | --- |
| Lysis Buffer | 100 mM HEPES pH 8.0, 500 mM NaCl, 10 % glycerol, 0.5 mM TCEP |
| IMAC Running Buffer | 20 mM HEPES pH 7.5, 500 mM NaCl, 10 % glycerol, 10 mM imidazole, 0.5 mM TCEP |
| IMAC Elution Buffer | 20 mM HEPES pH 7.5, 500 mM NaCl, 10 % glycerol, 500 mM imidazole, 0.5 mM TCEP |
| Size-exclusion Buffer | 20 mM HEPES pH 7.5, 300 mM NaCl, 10 % glycerol, 0.5 mM TCEP |

**Supplementary Table 2. List of inhibitor bound human MTH1 structures available in the Protein Data Bank.** Structures are listed in order of oldest to most recently solved. The RMSD values represent comparisons between the listed structures and hMTH1-Stz.

| PDB ID | Res (Å) | Ligand | IC50 | Structure | Reference | RMSD (Å) |
| --- | --- | --- | --- | --- | --- | --- |
| 3WHW | 2.70 | RUX | 6 nM | 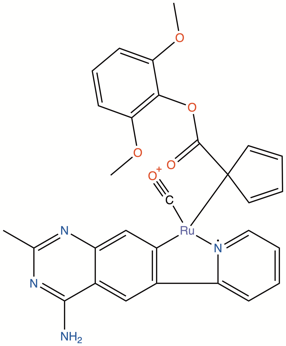 | [1] | 0.80 |
| 4C9X | 1.20 | VHS | 47 nM | 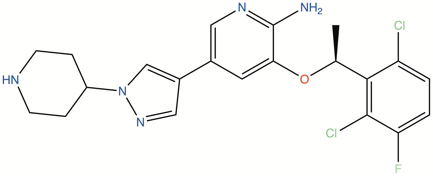 | [2] | 0.40 |
| 4C9W | 1.65 | VGH | 0.78 mM | 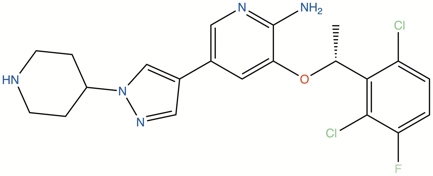 | [2] | 0.36 |
| 4N1U | 1.60 | 2GE | 5 nM | 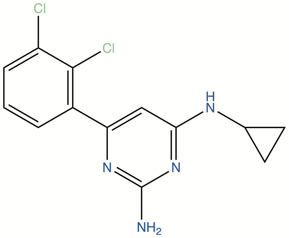 | [3] | 0.96 |
| 4N1T | 1.60 | 2GD | 0.8 nM | 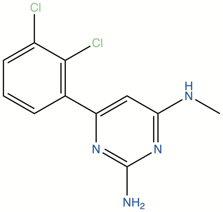 | [3] | 0.46 |
| 5ANW | 1.37 | 9CQ | 9 nM | 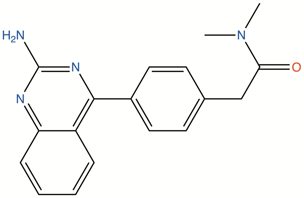 | [4] | 0.53 |
| 5ANV | 1.16 | RGJ | 0.9 nM | 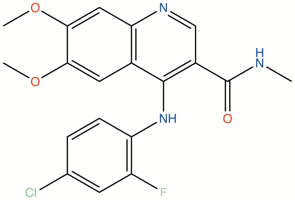 | [4] | 0.45 |
| 5ANU | 1.80 | 58T | 0.5 nM | 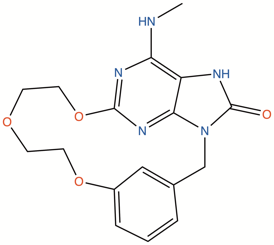 | [4] | 0.41 |
| 5ANT | 2.00 | RJE | 0.53 mM | 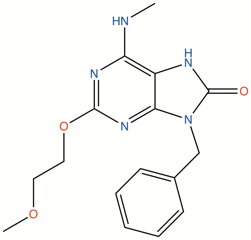 | [4] | 0.55 |
| 5ANS | 1.60 | RX8 | 2.05 mM | 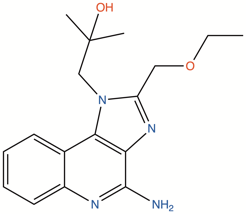 | [4] | 0.95 |
| 5FSN | 1.69 | 6Q3 | 4.5 mM | 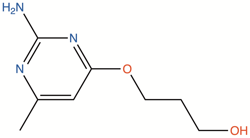 | [5] | 0.46 |
| 5FSM | 1.67 | N91 | >80 mM | 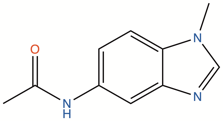 | [5] | 0.47 |
| 5FSL | 1.24 | UAN | 59 mM | 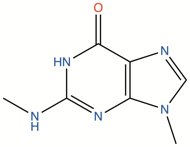 | [5] | 0.53 |
| 5FSO | 1.67 | S76 | 1.5 mM | 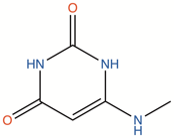 | [5] | 0.50 |
| 5NHY | 1.72 | 8XT | 2.3 nM | 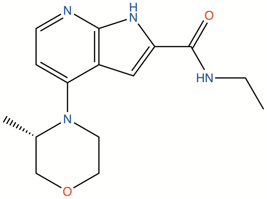 | [6] | 0.71 |
| 5NGT | 1.54 | 8WZ | 1.3 mM | 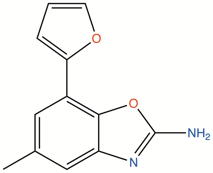 | [7] | 0.40 |
| 5NGS | 1.85 | 8WW | 0.17 mM | 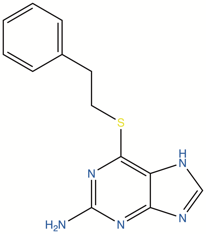 | [7] | 0.75 |
| 5NGR | 2.20 | 8WT | 24 mM | 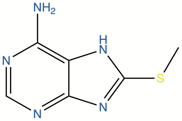 | [7] | 0.66 |
| 6F23 | 1.84 | C8Z | 5 nM | 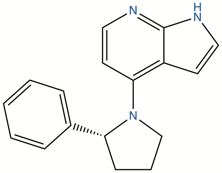 | [8] | 0.73 |
| 6F22 | 1.55 | C9B | 2 nM | 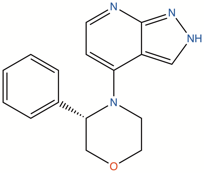 | [8] | 0.78 |
| 6F20 | 2.00 | C9E | 9.9 mM | 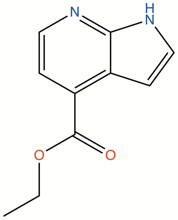 | [8] | 0.73 |
| 6F1X | 1.90 | C9Q | 20 nM | 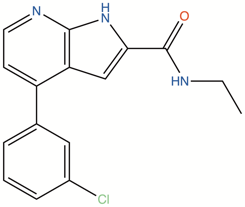 | [8] | 0.75 |
| 6EQ7 | 1.50 | BS8 | 30 nM | 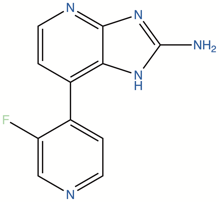 | [9] | 0.49 |
| 6EQ6 | 2.00 | EV2 | 23 mM | 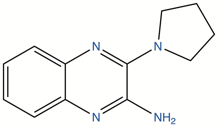 | [9] | 0.37 |
| 6EQ5 | 1.80 | AX7 | 44 mM | 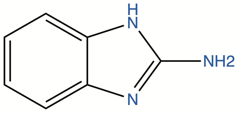 | [9] | 0.47 |
| 6EQ4 | 1.40 | BSW | 0.8 mM | 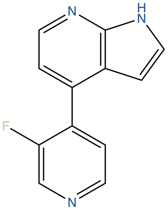 | [9] | 0.58 |
| 6EQ3 | 1.79 | BU5 | 0.4 mM | 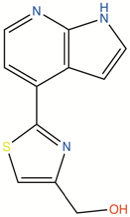 | [9] | 0.57 |
| 6EQ2 | 1.80 | BU8 | 1.1 mM | 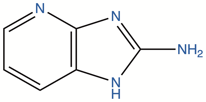 | [9] | 0.47 |
| 6GLV | 1.60 | F3E | - | 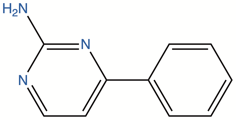 | Unpublished | 0.54 |
| 6GLU | 1.70 | BU8 | 1.1 mM | 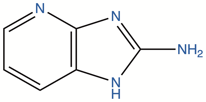 | Unpublished | 0.49 |
| 6GLS | 1.50 | F3E | - | 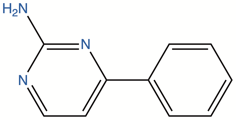 | Unpublished | 0.69 |
| 6GLR | 1.60 | F3E | - | 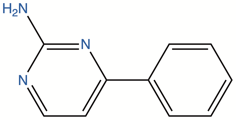 | Unpublished | 0.75 |
| 6GLQ | 1.60 | BU8 | 1.1 mM | 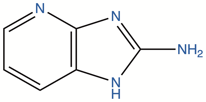 | Unpublished | 0.70 |
| 6GLP | 1.50 | BU8 | 1.1 mM | 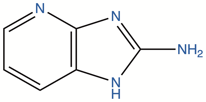 | Unpublished | 0.75 |
| 6GLN | 1.40 | F3E | - | 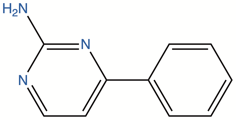 | Unpublished | 0.53 |
| 6GLM | 1.60 | BU8 | 1.1 mM | 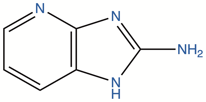 | Unpublished | 0.48 |
| 6GLL | 1.39 | BU8 | 1.1 mM | 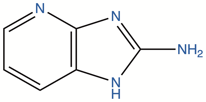 | Unpublished | 0.74 |
| 6GLJ | 1.30 | F3E | - | 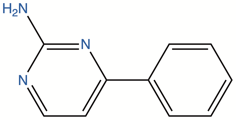 | Unpublished | 0.55 |
| 6GLI | 1.60 | F3E | - | 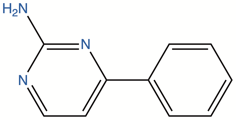 | Unpublished | 0.54 |
| 6GLH | 1.20 | BU8 | 1.1 mM | 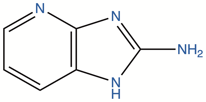 | Unpublished | 0.52 |
| 6GLG | 1.31 | BU8 | 1.1 mM | 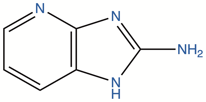 | Unpublished | 0.41 |
| 6GLE | 1.40 | F3E | - | 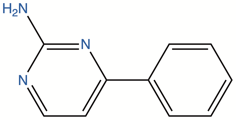 | Unpublished | 0.52 |
| 6AA5 | 1.90 | MKU | 52 nM | 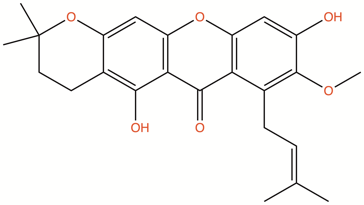 | [10] | 0.86 |
| 6AA4 | 1.90 | MKS | 470 nM | 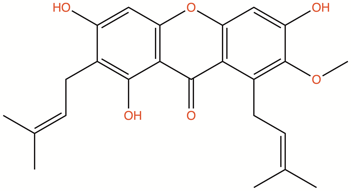 | [10] | 0.81 |
| 6IMZ | 2.10 | VGH | 0.78 mM | 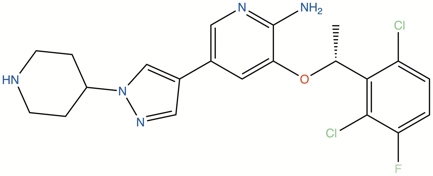 | Unpublished | 1.03 |
| 6US4 | 1.95 | GN6 | 13 nM | 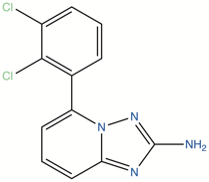 | [11] | 0.39 |
| 6US3 | 1.47 | 8JF | 81 nM | 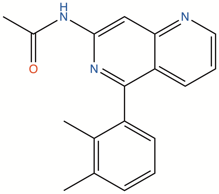 | [11] | 0.48 |
| 6US2 | 1.80 | S3O | 0.04 nM | 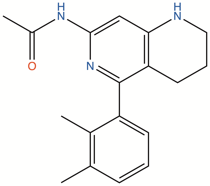 | [11] | 0.46 |
| 6JVT | 1.80 | CJR | 0.14 mM | 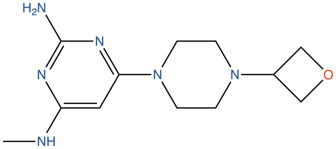 | [12] | 0.76 |
| 6JVS | 2.10 | CLJ | 2.0 mM | 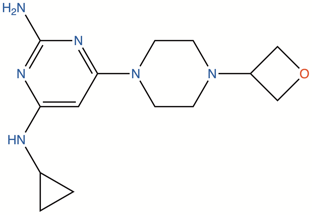 | [12] | 0.79 |
| 6JVR | 2.29 | CJU | 2.2 nM | 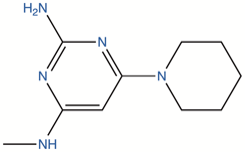 | [12] | 0.77 |
| 6JVQ | 2.19 | CJF | 41 nM | 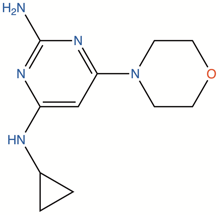 | [12] | 0.79 |
| 6JVP | 2.20 | CJ9 | 6 nM | 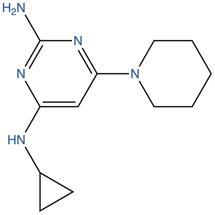 | [12] | 0.82 |
| 6JVO | 1.90 | CJ6 | 1.5 mM | 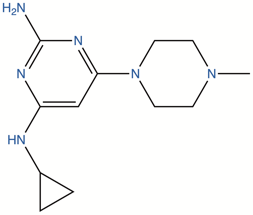 | [12] | 0.72 |
| 6JVN | 2.10 | CJ0 | 10 nM | 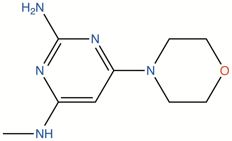 | [12] | 0.75 |
| 6JVM | 2.09 | CGX | 4.8 mM | 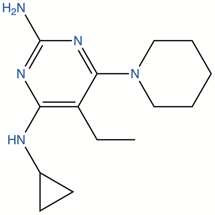 | [12] | 0.75 |
| 6JVL | 1.90 | CG0 | >1 mM | 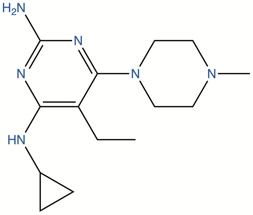 | [12] | 0.75 |
| 6JVK | 2.10 | CEU | 0.31 mM | 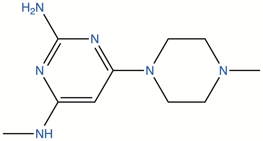 | [12] | 0.72 |
| 6JVJ | 2.29 | C9L | 71.1 mM | 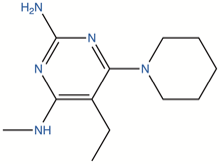 | [12] | 0.74 |
| 6JVI | 2.24 | 95R | 14 mM | 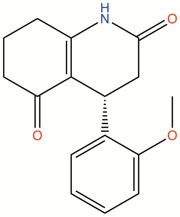 | [12] | 0.68 |
| 6JVH | 2.04 | 95L | 1.2 mM | 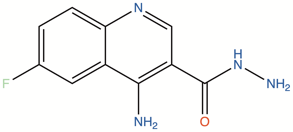 | [12] | 0.74 |
| 6JVG | 1.84 | 95F | 6.3 mM | 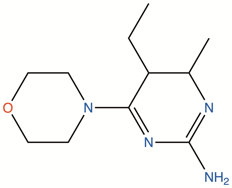 | [12] | 0.73 |
| 7N13 | 1.59 | ZRV | n.a. | 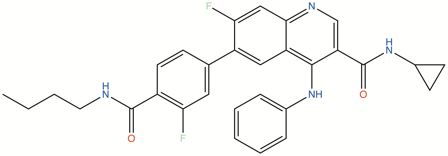 | [13] | 0.75 |
| 7N03 | 1.13 | ZRP | n.a. | 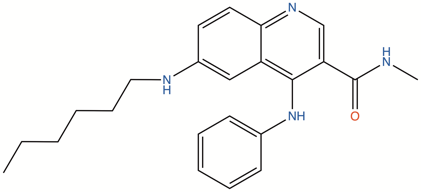 | [13] | 0.49 |
| 8A0T | 1.90 | KLO | n.a. | 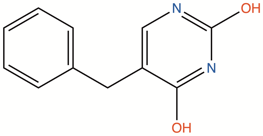 | Unpublished | 0.76 |
| 8A07 | 2.19 | L3N | n.a. | 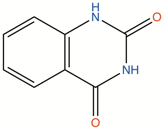 | Unpublished | 0.78 |
| 8A3A | 1.60 | KYR | n.a. | 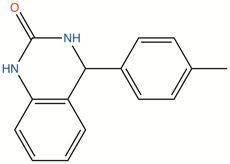 | Unpublished | 0.51 |
|  |  |  |  |  |  |  |
| 8A0S | 1.40 | KOX | n.a. | 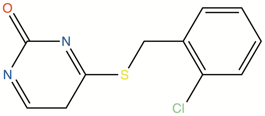 | Unpublished | 0.56 |
| 8A34 | 1.90 | KYI | n.a. | 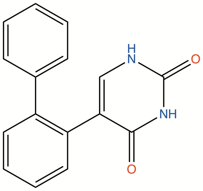 | Unpublished | 0.67 |

**Supplementary Table 3. List of human proteins structures in complex with DHT available in the Protein Data Bank.** The full name of the protein is stated in the first instance, and thereafter only the protein acronym is shown.

| PDB ID | Res (Å) | Protein name | Reference |
| --- | --- | --- | --- |
| 1DHT | 2.24 | 17-β-hydroxysteroid dehydrogenase type 1 (17β-HSD1) | [14] |
| 1D2S | 1.55 | Sex hormone-binding globulin (SHBG) | [15] |
| 1F5F | 1.70 | SHBG | [16] |
| 1KDM | 2.35 | SHBG | [17] |
| 1KDK | 1.70 | SHBG | [17] |
| 1XJ7 | 2.70 | Androgen receptor ligand-binding domain (AR-LBD) | [18] |
| 1T65 | 1.66 | AR-LBD | [18] |
| 1T63 | 2.07 | AR-LBD | [18] |
| 1T5Z | 2.30 | AR-LBD | [18] |
| 2AMA | 1.90 | AR-LBD | [19] |
| 2PKL | 2.49 | AR-LBD | [20] |
| 2PIX | 2.40 | AR-LBD | [20] |
| 2PIW | 2.58 | AR-LBD | [20] |
| 2P1V | 1.95 | AR-LBD | [20] |
| 2PIU | 2.12 | AR-LBD | [20] |
| 2PIT | 1.76 | AR-LBD | [20] |
| 2PIR | 2.10 | AR-LBD | [20] |
| 2PIQ | 2.40 | AR-LBD | [20] |
| 2PIP | 1.80 | AR-LBD | [20] |
| 2PIO | 2.03 | AR-LBD | [20] |
| 2Z4J | 2.60 | AR-LBD | [21] |
| 3DEY | 1.70 | AR-LBD | Unpublished |
| 3L3Z | 2.00 | AR-LBD | [22] |
| 3L3X | 1.55 | AR-LBD | [22] |
| 3KLM | 1.70 | 17β-HSD1 | [23] |
| 4K7A | 2.44 | AR-LBD | Unpublished |
| 4OFU | 2.12 | AR-LBD | [24] |
| 4OFR | 2.26 | AR-LBD | [24] |
| 4OEZ | 1.80 | AR-LBD | [24] |
| 4OEY | 1.83 | AR-LBD | [24] |
| 4OED | 2.79 | AR-LBD | [24] |
| 4OEA | 2.12 | AR-LBD | [24] |
| 5JJM | 2.15 | AR-LBD | [25] |
| 7ZU2 | 1.74 | AR-LBD (Q799E) | [26] |
| 7ZU1 | 1.68 | AR-LBD (V758A) | [26] |
| 7ZTZ | 1.40 | AR-LBD (Y764C) | [26] |
| 7ZTX | 1.89 | AR-LBD (F755V) | [26] |
| 7ZTV | 1.94 | AR-LBD (F755L) | [26] |
| 8FH2 | 1.59 | AR-LBD (L702H/H875Y) | [27] |
| 8FH1 | 1.69 | AR-LBD (F877L/T878A) | [27] |
| 8FH0 | 1.59 | AR-LBD (H875Y/F877L/T878A) | [27] |
| 8FGZ | 1.61 | AR-LBD (L702H/H875Y/F877L) | [27] |
| 8FGY | 2.20 | AR-LBD (L702H/H875Y/F877L/T878A) | [27] |

Structures are listed in order of oldest to most recently solved.

**Supplementary Table 4. List of nucleotide bound human MTH1 available in the Protein Data Bank.** Structures are listed in order of oldest to most recently solved. The RMSD values represent comparisons between the listed structures and hMTH1-Stz.

| PDB ID | Res (Å) | Nucleotide | Structure | Reference | RMSD (Å) |
| --- | --- | --- | --- | --- | --- |
| 3ZR0 | 1.80 | 8-oxo-dGMP | 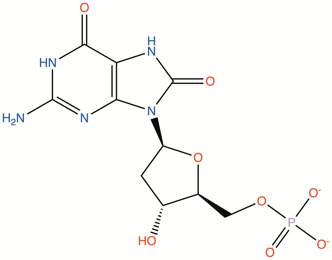 | [28] | 0.77 |
| 5FSI | 1.63 | 8-oxo-dGTP | 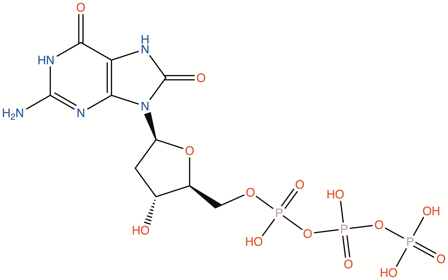 | [5] | 0.52 |
| 5FSK | 1.56 | 8-oxo-ATP | 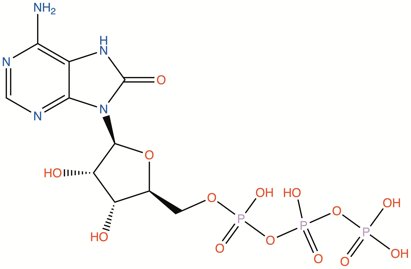 | [5] | 0.49 |
| 5WS7 | 1.00 | 2-oxo-dATP | 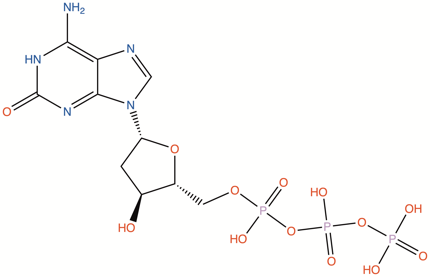 | [29] | 0.85 |
| 5GHQ | 1.18 | 2-oxo-dATP | 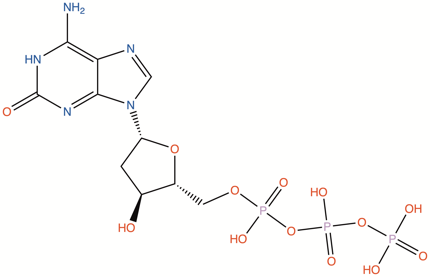 | [29] | 0.62 |
| 5GHO | 1.19 | 8-oxo-dGTP | 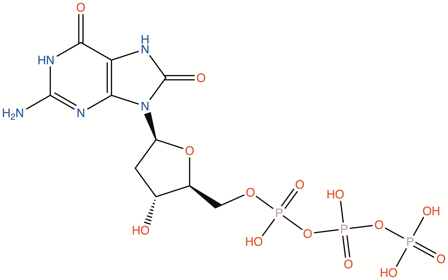 | [29] | 0.66 |
| 5GHN | 1.39 | 2-oxo-dATP | 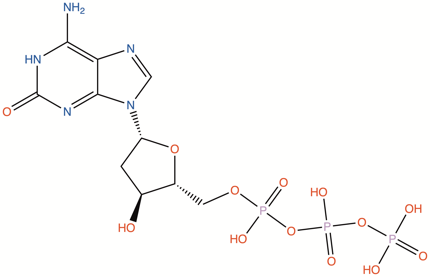 | [29] | 0.69 |
| 5GHM | 1.50 | 8-oxo-dGTP | 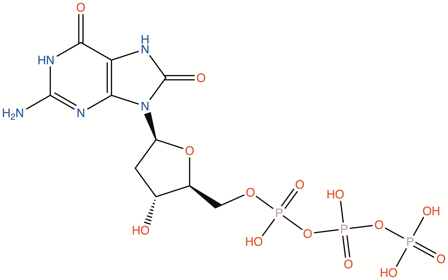 | [29] | 0.69 |
| 5GHJ | 1.20 | 2-oxo-dATP | 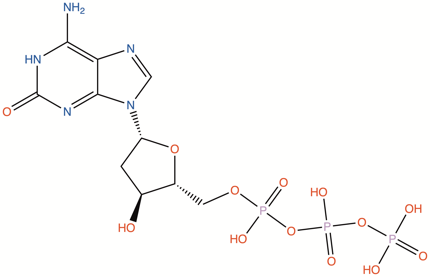 | [29] | 0.64 |
| 5GHI | 1.21 | 8-oxo-dGTP | 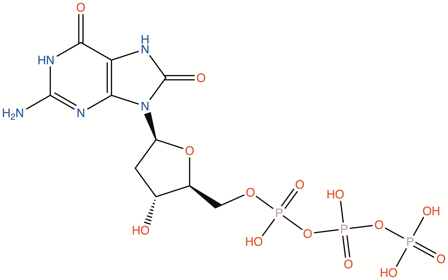 | [29] | 0.79 |
| 6ILI | 1.45 | 8-oxo-dGTP | 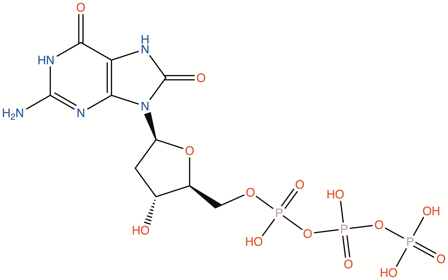 | [29] | 0.72 |
| 5OTM | 1.80 | O6-methyl-dGMP | 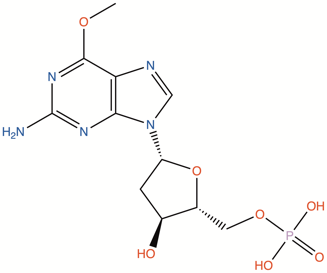 | [30] | 0.76 |
| 6IJY | 1.04 | 8-oxo-dGTP | 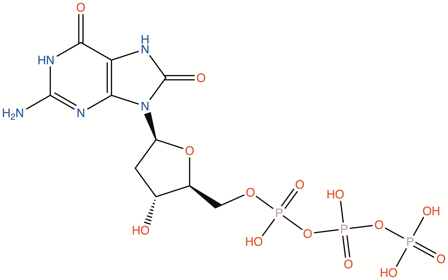 | [31] | 0.74 |
| 6QVO | 2.45 | N6-methyl-dAMP | 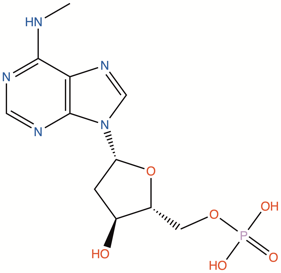 | [32] | 0.72 |
| 8I8T | 1.22 | 2-oxo-dAMP | 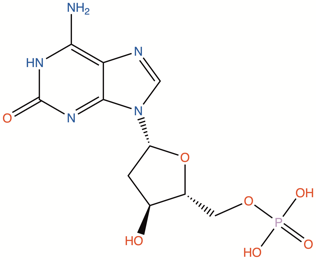 | [33] | 0.78 |
| 8I8S | 1.42 | 8-oxo-dGMP | 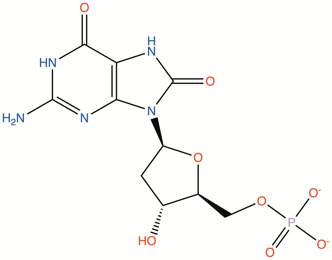 | [33] | 0.76 |
| 8I1J | 1.08 | 2-oxo-dATP | 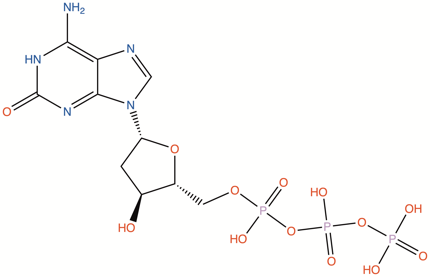 | [33] | 0.70 |
| 8I1I | 1.20 | 2-oxo-dATP | 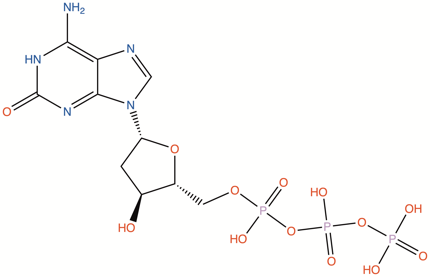 | [33] | 0.69 |
| 8I1G | 1.18 | 2-oxo-dATP | 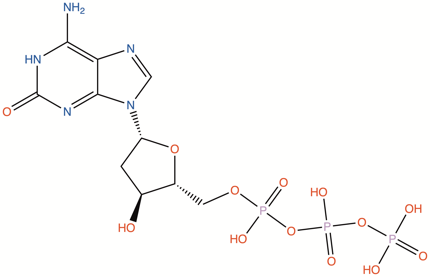 | [33] | 0.62 |
| 8I1F | 1.05 | 2-oxo-dATP | 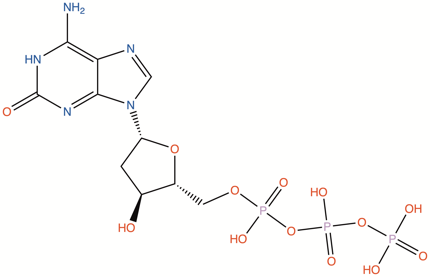 | [33] | 0.61 |
| 8I1E | 1.10 | 2-oxo-dATP | 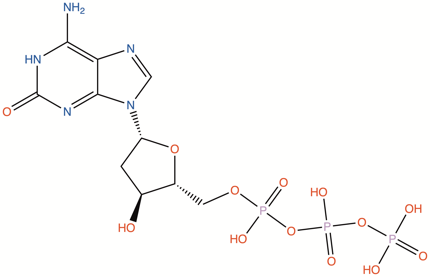 | [33] | 0.71 |
| 8I1D | 1.20 | 2-oxo-dATP | 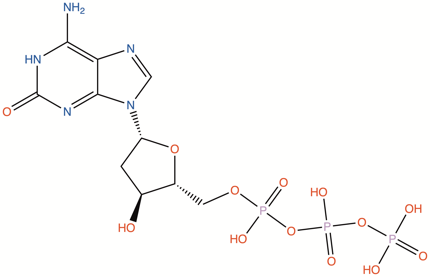 | [33] | 0.58 |
| 8I1A | 1.40 | 8-oxo-dGTP | 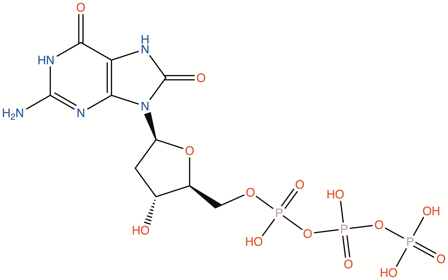 | [33] | 0.62 |
| 8I19 | 1.48 | 8-oxo-dGTP | 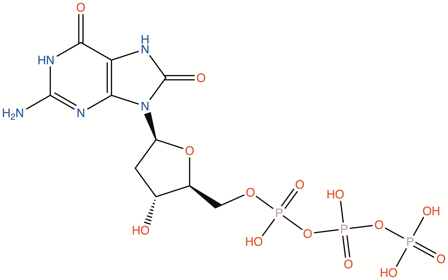 | [33] | 0.79 |
| 8I18 | 1.10 | 8-oxo-dGTP | 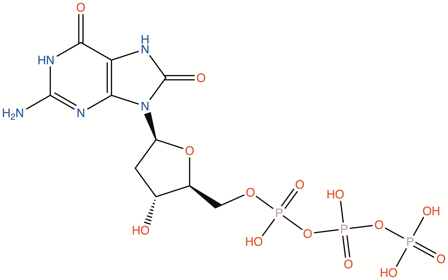 | [33] | 0.81 |

Structures are listed in order of oldest to most recently solved.


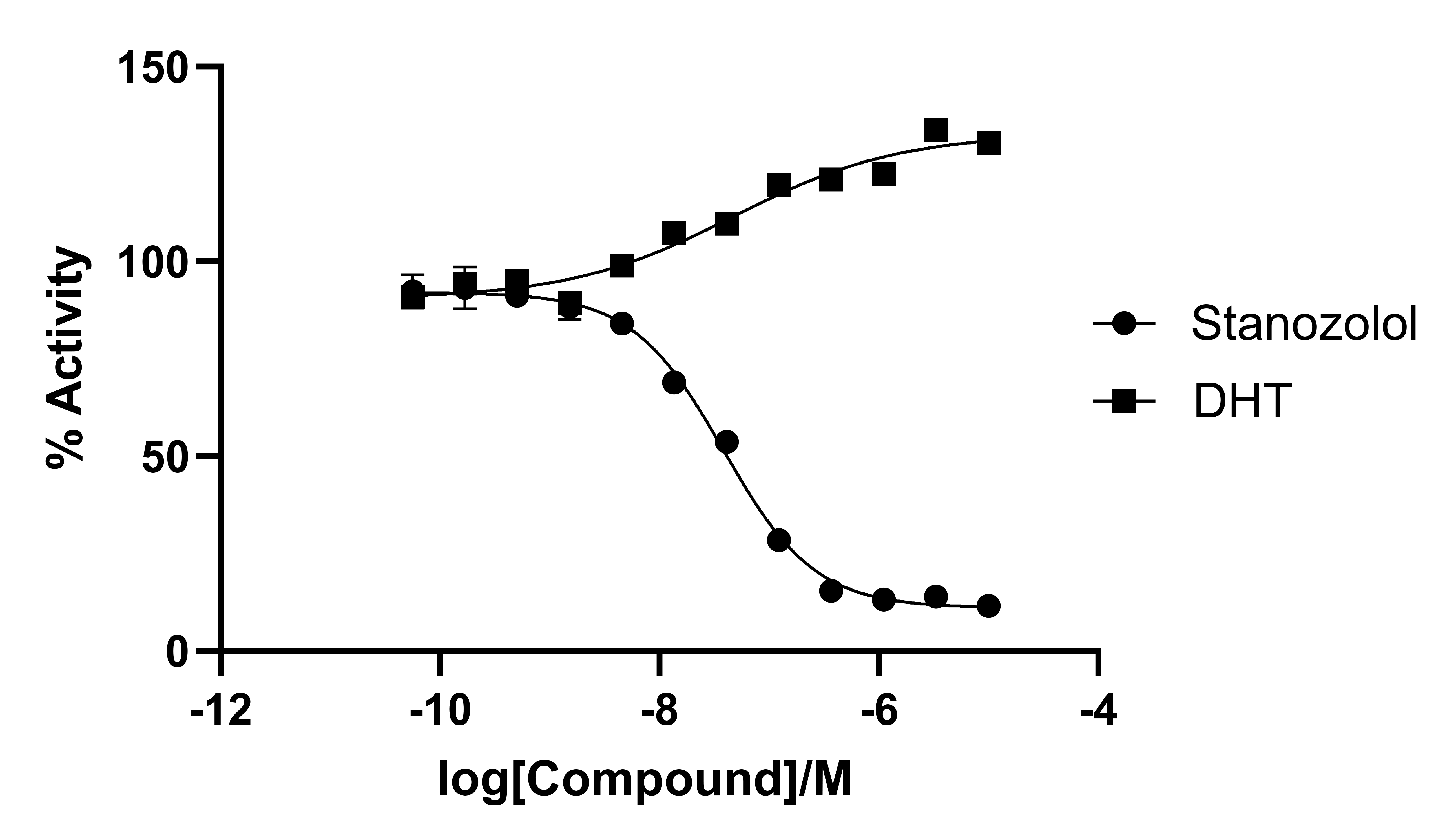


**Supplementary Figure 1. Stanozolol and DHT dose-response curves for human MTH1.** In these experiments, dGTP was used as the assay substrate. Data are shown as mean ± standard deviation (SD). Refer to Materials and methods section for details on experimental procedures.


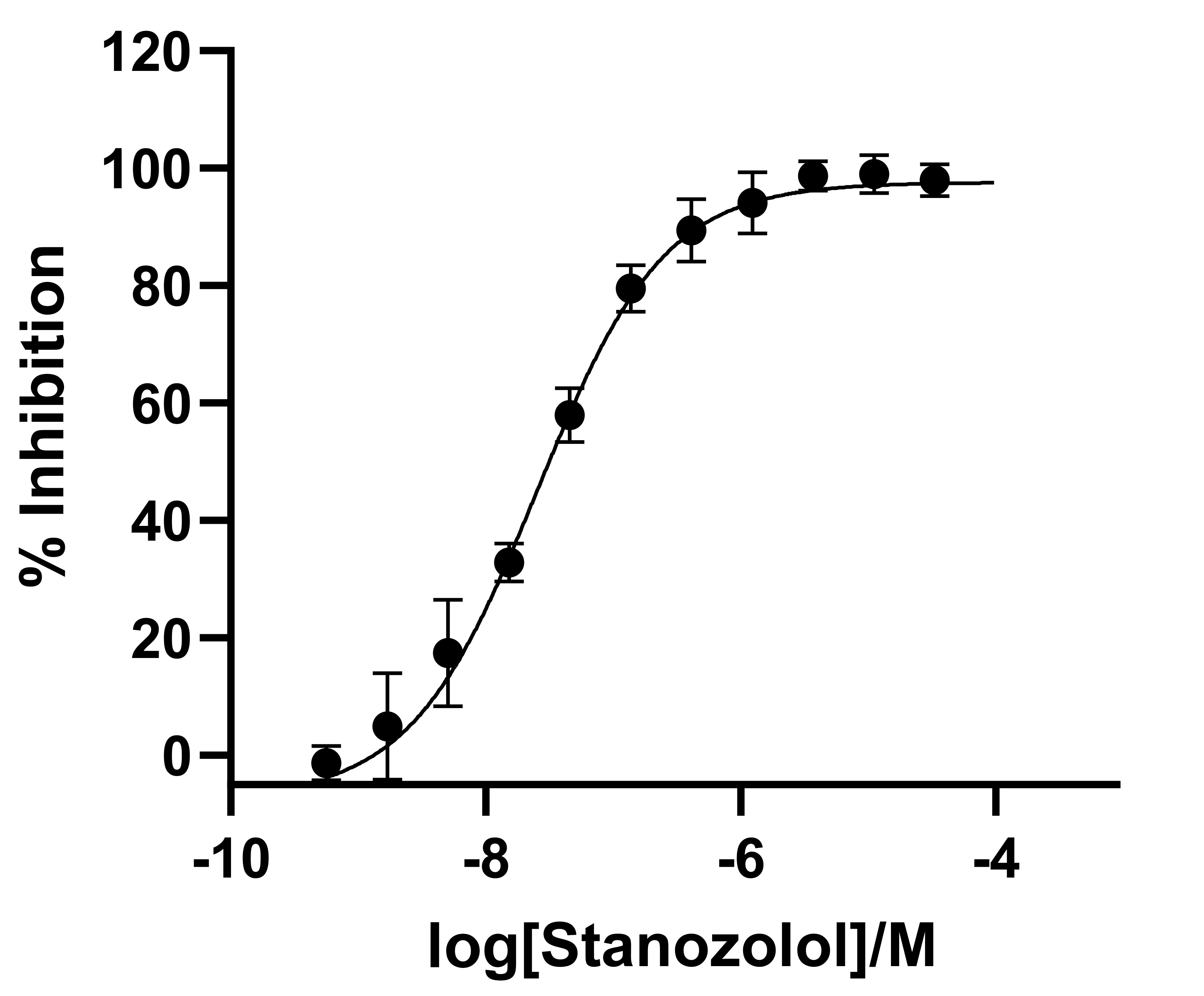


**Supplementary Figure 2. Stanozolol dose-response curves for human MTH1.** In this experiment, 8-oxo-dGTP was used as the assay substrate. IC50=30 nM (n=3). Data are shown as mean ± standard deviation (SD). Refer to Materials and methods section for details on experimental procedures.


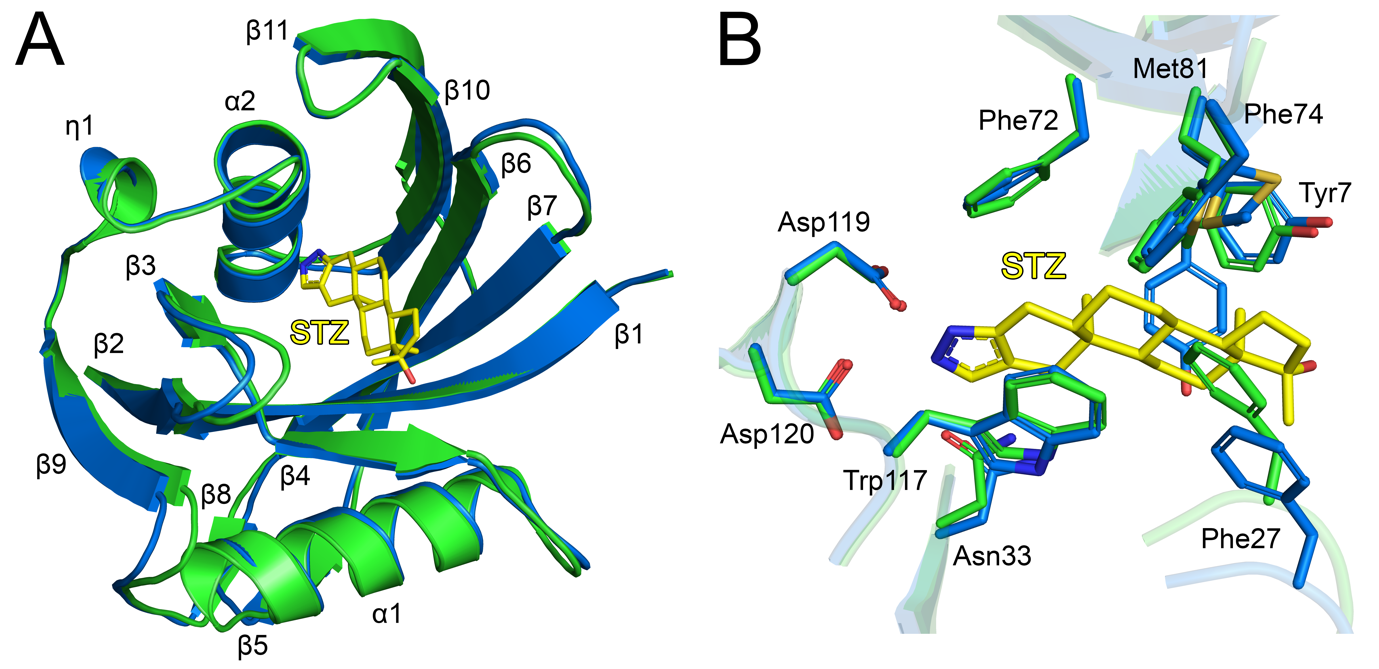


**Supplementary Figure 3. Comparison of stanozolol bound hMTH1 and the hMTH1 apo structure.** (A) Cα-atom superposition of whole monomers visualized as ribbon representations. The hMTH1-Stz structure is coloured blue and apo hMTH1 (PDB ID: 3ZR1) is coloured green. The Stz ligand is depicted as a stick model; C atoms coloured yellow, O atoms red and N atoms dark blue. The secondary structure elements, α-helices (α1-2), β-strands (β1-11) and 3_10_-helices (η1) are labelled. (B) Comparison of the active site region of hMTH1-Stz with apo hMTH1. Amino acids are depicted as sticks; C atoms are coloured blue (hMTH1-Stz) or green (apo hMTH1), O atoms red, N atoms dark blue and S atoms gold. Figure produced with PyMOL (version 3.0.4, Schrödinger).


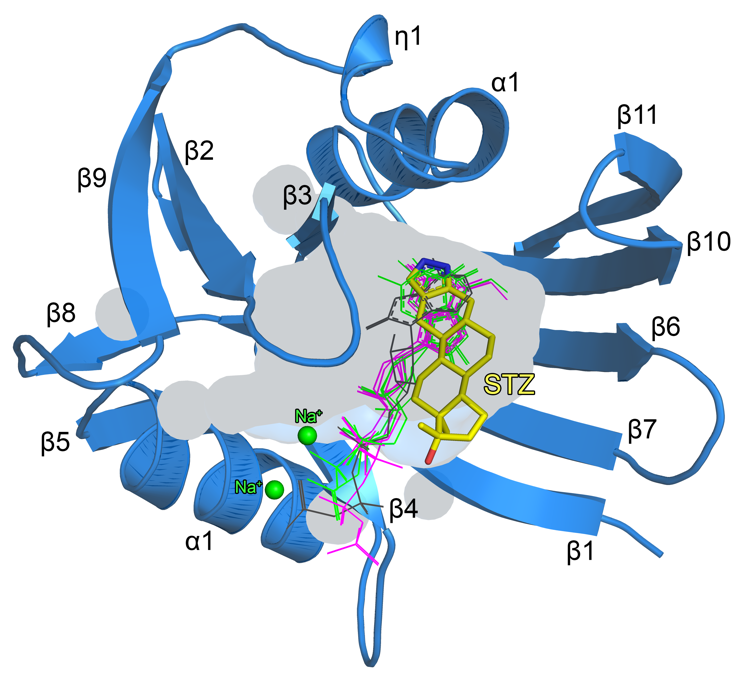


**Supplementary Figure 4. Comparison of hMTH1-Stanozolol complex with hMTH1 nucleotide bound structures.** The Cα-atoms of hMTH1-Stz were superimposed with hMTH1 structures bound with 8-oxo-dGMP (PDB ID: 8I8S), 8-oxo-dGTP (PDB ID: 5GHI), 8-oxo-ATP (PDB ID: 5FSK), 2-oxo-dATP (PDB ID: 5WS7), O6-methyl-dGMP (PDB ID: 5OTM), N6-methyl-dAMP (PDB ID: 6QVO) and 2-oxo-dAMP (PDB ID: 8I8T). For clarity, only the monomer of hMTH1-Stz is shown. The ligand binding cavity of hMTH1-Stz is shown as a semi-transparent surface. Stz is depicted as a stick model; C atoms coloured yellow, O atoms red and N atoms dark blue. The nucleotide substrates from the other hMTH1 structures are shown as either green (8-oxo-dGMP, 8-oxo-dGTP, O6-methyl-dGMP), dark grey (8-oxo-ATP) or magenta (2-oxo-dATP, N6-methyl-dAMP, 2-oxo-dAMP) lines. Two sodium ions from the 8-oxo-dGTP bound structure are shown as green spheres. The secondary structure elements, α-helices (α1-2), β-strands (β1-11) and 3_10_-helices (η1) are labelled. Figure produced with PyMOL (version 3.0.4, Schrödinger).

**
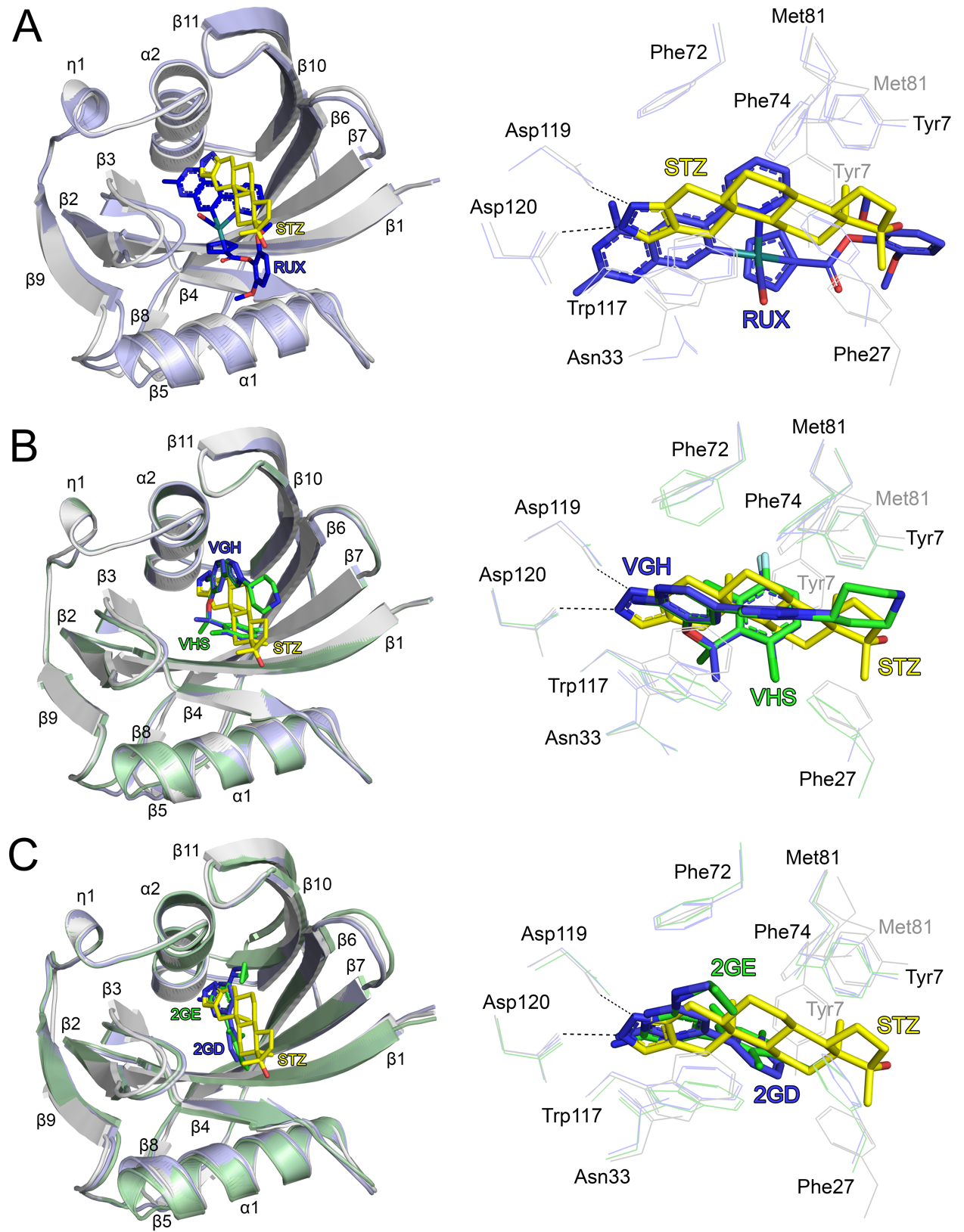
**

**Supplementary Figure 5. Comparison of stanozolol bound hMTH1 and the hMTH1 inhibitor bound structures from Streib *et al*, Huber *et al* and Gad *et al*.** *Left*: Cα-atom superpositions of whole monomers visualized as ribbon representations. The secondary structure elements, α-helices (α1-2), β-strands (β1-11) and 3_10_-helices (η1) are labelled. *Right*: Comparison of the active site region of hMTH1-Stz with inhibitor bound hMTH1 structures. In all panels the hMTH1-Stz structure is coloured light grey. The Stz ligand is depicted as a stick model; C atoms coloured yellow, O atoms red and N atoms dark blue. Hydrogen bond interactions are shown as dashed lines. The alternative conformations of Tyr7 and Met81 in hMTH1-Stz are labelled in grey. **(A)** Comparison with Streib *et al*., structures [1]. hMTH1-RUX (PDB ID: 3WHW) is coloured blue. **(B)** Comparison with Huber *et al*., structures [2]. hMTH1-VGH (PDB ID: 4C9W) is coloured blue and hMTH1-VHS (PDB ID: 4C9X) is coloured green. **(C)** Comparison with Gad *et al*., structures [3]. hMTH1-2GD (PDB ID: 4N1T) is coloured blue and hMTH1-2GE (PDB ID: 4N1U) is coloured green. Figure produced with PyMOL (version 3.0.4, Schrödinger).


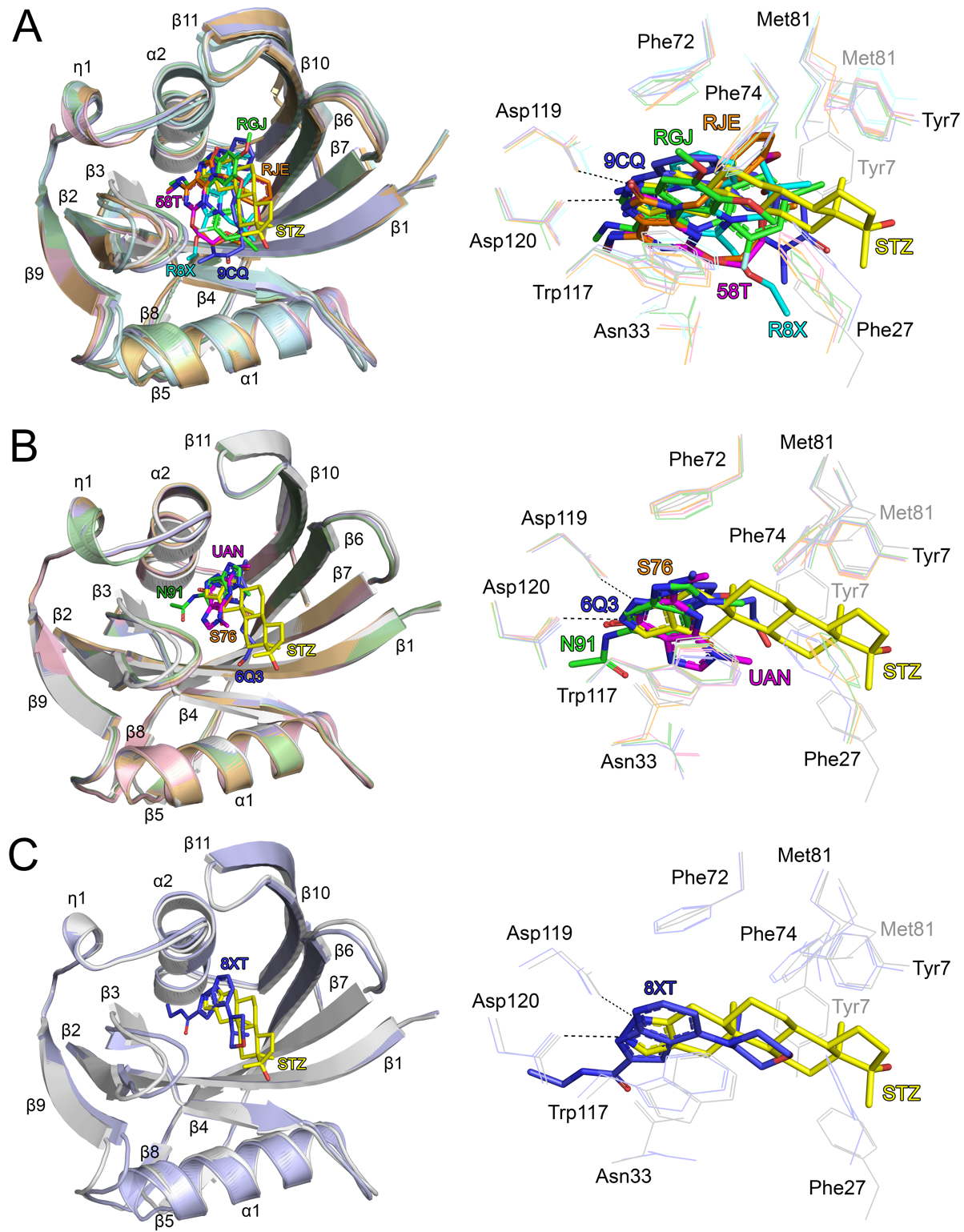


**Supplementary Figure 6. Comparison of stanozolol bound hMTH1 and the hMTH1 inhibitor bound structures from Kettle *et al*, Nissink *et al* and Ellermann *et al*.** *Left*: Cα-atom superpositions of whole monomers visualized as ribbon representations. The secondary structure elements, α-helices (α1-2), β-strands (β1-11) and 3_10_-helices (η1) are labelled. *Right*: Comparison of the active site region of hMTH1-Stz with inhibitor bound hMTH1 structures. In all panels the hMTH1-Stz structure is coloured light grey. The Stz ligand is depicted as a stick model; C atoms coloured yellow, O atoms red and N atoms dark blue. Hydrogen bond interactions are shown as dashed lines. The alternative conformations of Tyr7 and Met81 in hMTH1-Stz are labelled in grey. **(A)** Comparison with Kettle *et al*., structures [4]. hMTH1-9CQ (PDB ID: 5ANW) is coloured blue, hMTH1-RGJ (PDB ID: 5ANV) is coloured green, hMTH1-58T (PDB ID: 5ANU) is coloured pink, hMTH1-RJE (PDB ID: 5ANT) is coloured orange and hMTH1-R8X (PDB ID: 5ANS) is coloured cyan. **(B)** Comparison with Nissink *et al*., structures [5]. hMTH1-6Q3 (PDB ID: 5FSN) is coloured blue, hMTH1-N91 (PDB ID: 5FSM) is coloured green, hMTH1-UAN (PDB ID: 5FSL) is coloured pink and hMTH1-S76 (PDB ID: 5FSO) is coloured orange. **(C)** Comparison with Ellermann *et al*., structure [6]. hMTH1-8XT (PDB ID: 5NHY) is coloured blue. Figure produced with PyMOL (version 3.0.4, Schrödinger).

**Supplementary Figure 7. Comparison of stanozolol bound hMTH1 and the hMTH1 inhibitor bound structures from Rudling *et al*, Rahm *et al* and Wiedmer *et al*.** *Left*: Cα-atom superpositions of whole monomers visualized as ribbon representations. The secondary structure elements, α-helices (α1-2), β-strands (β1-11) and 3_10_-helices (η1) are labelled. *Right*: Comparison of the active site region of hMTH1-Stz with inhibitor bound hMTH1 structures. In all panels the hMTH1-Stz structure is coloured light grey. The Stz ligand is depicted as a stick model; C atoms coloured yellow, O atoms red and N atoms dark blue. Hydrogen bond interactions are shown as dashed lines. The alternative conformations of Tyr7 and Met81 in hMTH1-Stz are labelled in grey. **(A)** Comparison with Rudling *et al*., structures [7]. hMTH1-8WZ (PDB ID: 5NGT) is coloured blue, hMTH1-8WW (PDB ID: 5NGS) is coloured green and hMTH1-8WT (PDB ID: 5NGR) is coloured pink, **(B)** Comparison with Rahm *et al*., structures [8]. hMTH1-C8Z (PDB ID: 6F23) is coloured blue, hMTH1-C9B (PDB ID: 6F22) is coloured green, hMTH1-C9E (PDB ID: 6F20) is coloured pink and hMTH1-C9Q (PDB ID: 6F1X) is coloured orange. **(C)** Comparison with Wiedmer *et al*., structures [9]. hMTH1-BS8 (PDB ID: 6EQ7) is coloured blue, hMTH1-EV2 (PDB ID: 6EQ6) is coloured green, hMTH1-AX7 (PDB ID: 6EQ5) is coloured pink, hMTH1-BSW (PDB ID: 6EQ4) is coloured orange, hMTH1-BU5 (PDB ID: 6EQ3) is coloured cyan and hMTH1-BU8 (PDB ID: 6EQ2) is coloured purple. Figure produced with PyMOL (version 3.0.4, Schrödinger).

**Supplementary Figure 8. Comparison of stanozolol bound hMTH1 and the hMTH1 inhibitor bound structures from Yokoyama *et al*, Farand *et al* and Veits *et al*.** *Left*: Cα-atom superpositions of whole monomers visualized as ribbon representations. The secondary structure elements, α-helices (α1-2), β-strands (β1-11) and 3_10_-helices (η1) are labelled. *Right*: Comparison of the active site region of hMTH1-Stz with inhibitor bound hMTH1 structures. In all panels the hMTH1-Stz structure is coloured light grey. The Stz ligand is depicted as a stick model; C atoms coloured yellow, O atoms red and N atoms dark blue. Hydrogen bond interactions are shown as dashed lines. The alternative conformations of Tyr7 and Met81 in hMTH1-Stz are labelled in grey. **(A)** Comparison with Yokoyama *et al*., structures [10]. hMTH1-MKU (PDB ID: 6AA5) is coloured blue and hMTH1-MKS (PDB ID: 6AA4) is coloured green **(B)** Comparison with Farand *et al*., structures [11]. hMTH1-GN6 (PDB ID: 6US4) is coloured blue, hMTH1-8JF (PDB ID: 6US3) is coloured green and hMTH1-S3O (PDB ID: 6US2) is coloured pink. **(C)** Comparison with Veits *et al*., structures [13]. hMTH1-ZRV (PDB ID: 7N13) is coloured blue and hMTH1-ZRP (PDB ID: 7N03) is coloured green. Figure produced with PyMOL (version 3.0.4, Schrödinger).

**Supplementary Figure 9. Comparison of stanozolol bound hMTH1 and the hMTH1 inhibitor bound structures from Peng *et al.* and unpublished structures.** *Left*: Cα-atom superpositions of whole monomers visualized as ribbon representations. The secondary structure elements, α-helices (α1-2), β-strands (β1-11) and 3_10_-helices (η1) are labelled. *Right*: Comparison of the active site region of hMTH1-Stz with inhibitor bound hMTH1 structures. In all panels the hMTH1-Stz structure is coloured light grey. The Stz ligand is depicted as a stick model; C atoms coloured yellow, O atoms red and N atoms dark blue. Hydrogen bond interactions are shown as dashed lines. The alternative conformations of Tyr7 and Met81 in hMTH1-Stz are labelled in grey. **(A)** Comparison with Peng *et al*., structures [12]. hMTH1-CJR (PDB ID: 6JVT) is coloured blue, hMTH1-CLJ (PDB ID: 6JVS) is coloured green, hMTH1-CJU (PDB ID: 6JVR) is coloured pink, hMTH1-CJF (PDB ID: 6JVQ) is coloured orange, hMTH1-CJ9 (PDB ID: 6JVP) is coloured cyan, hMTH1-CJ6 (PDB ID: 6JVO) is coloured purple and hMTH1-CJ0 (PDB ID: 6JVN) is coloured brown. **(B)** Comparison with Peng *et al*., structures [12]. hMTH1-CGX (PDB ID: 6JVM) is coloured blue, hMTH1-CG0 (PDB ID: 6JVL) is coloured green, hMTH1-CEU (PDB ID: 6JVK) is coloured pink, hMTH1-CL9 (PDB ID: 6JVJ) is coloured orange, hMTH1-95R (PDB ID: 6JVI) is coloured cyan, hMTH1-95L (PDB ID: 6JVH) is coloured purple and hMTH1-95F (PDB ID: 6JVG) is coloured brown. **(C)** Comparison with unpublished PDB structures. hMTH1-F3E (PDB ID: 6GLE) is coloured blue, hMTH1-KLO (PDB ID: 8A0T) is coloured green, hMTH1-L3N (PDB ID: 8A07) is coloured pink, hMTH1-KYR (PDB ID: 8A3A) is coloured orange, hMTH1-KOX (PDB ID: 8A0S) is coloured cyan and hMTH1-KYI (PDB ID: 8A34) is coloured purple. Figure produced with PyMOL (version 3.0.4, Schrödinger)

**References**

1. Streib, M., Kräling, K., Richter, K., Xie, X., Steuber, H. & Meggers, E. (2014) An organometallic inhibitor for the human repair enzyme 7,8-dihydro-8-oxoguanosine triphosphatase, *Angew Chem Int Ed Engl.* **53**, 305-9.

2. Huber, K. V., Salah, E., Radic, B., Gridling, M., Elkins, J. M., Stukalov, A., Jemth, A. S., Göktürk, C., Sanjiv, K., Strömberg, K., Pham, T., Berglund, U. W., Colinge, J., Bennett, K. L., Loizou, J. I., Helleday, T., Knapp, S. & Superti-Furga, G. (2014) Stereospecific targeting of MTH1 by (S)-crizotinib as an anticancer strategy, *Nature.* **508**, 222-7.

3. Gad, H., Koolmeister, T., Jemth, A.-S., Eshtad, S., Jacques, S. A., Ström, C. E., Svensson, L. M., Schultz, N., Lundbäck, T. & Einarsdottir, B. O. (2014) MTH1 inhibition eradicates cancer by preventing sanitation of the dNTP pool, *Nature.* **508**, 215.

4. Kettle, J. G., Alwan, H., Bista, M., Breed, J., Davies, N. L., Eckersley, K., Fillery, S., Foote, K. M., Goodwin, L., Jones, D. R., Käck, H., Lau, A., Nissink, J. W., Read, J., Scott, J. S., Taylor, B., Walker, G., Wissler, L. & Wylot, M. (2016) Potent and Selective Inhibitors of MTH1 Probe Its Role in Cancer Cell Survival, *J Med Chem.* **59**, 2346-61.

5. Nissink, J. W. M., Bista, M., Breed, J., Carter, N., Embrey, K., Read, J. & Winter-Holt, J. J. (2016) MTH1 Substrate Recognition-An Example of Specific Promiscuity, *Plos One.* **11**.

6. Ellermann, M., Eheim, A., Rahm, F., Viklund, J., Guenther, J., Andersson, M., Ericsson, U., Forsblom, R., Ginman, T., Lindström, J., Silvander, C., Trésaugues, L., Giese, A., Bunse, S., Neuhaus, R., Weiske, J., Quanz, M., Glasauer, A., Nowak-Reppel, K., Bader, B., Irlbacher, H., Meyer, H., Queisser, N., Bauser, M., Haegebarth, A. & Gorjánácz, M. (2017) Novel Class of Potent and Cellularly Active Inhibitors Devalidates MTH1 as Broad-Spectrum Cancer Target, *ACS Chem Biol.* **12**, 1986-1992.

7. Rudling, A., Gustafsson, R., Almlof, I., Homan, E., Scobie, M., Warpman Berglund, U., Helleday, T., Stenmark, P. & Carlsson, J. (2017) Fragment-Based Discovery and Optimization of Enzyme Inhibitors by Docking of Commercial Chemical Space, *J Med Chem.* **60**, 8160-8169.

8. Rahm, F., Viklund, J., Trésaugues, L., Ellermann, M., Giese, A., Ericsson, U., Forsblom, R., Ginman, T., Günther, J., Hallberg, K., Lindström, J., Persson, L. B., Silvander, C., Talagas, A., Díaz-Sáez, L., Fedorov, O., Huber, K. V. M., Panagakou, I., Siejka, P., Gorjánácz, M., Bauser, M. & Andersson, M. (2018) Creation of a Novel Class of Potent and Selective MutT Homologue 1 (MTH1) Inhibitors Using Fragment-Based Screening and Structure-Based Drug Design, *J Med Chem.* **61**, 2533-2551.

9. Wiedmer, L., Schärer, C., Spiliotopoulos, D., Hürzeler, M., Śledź, P. & Caflisch, A. (2019) Ligand retargeting by binding site analogy, *European Journal of Medicinal Chemistry.* **175**, 107-113.

10. Yokoyama, T., Kitakami, R. & Mizuguchi, M. (2019) Discovery of a new class of MTH1 inhibitor by X-ray crystallographic screening, *European Journal of Medicinal Chemistry.* **167**, 153-160.

11. Farand, J., Kropf, J. E., Blomgren, P., Xu, J., Schmitt, A. C., Newby, Z. E., Wang, T., Murakami, E., Barauskas, O., Sudhamsu, J., Feng, J. Y., Niedziela-Majka, A., Schultz, B. E., Schwartz, K., Viatchenko-Karpinski, S., Kornyeyev, D., Kashishian, A., Fan, P., Chen, X., Lansdon, E. B., Ports, M. O., Currie, K. S., Watkins, W. J. & Notte, G. T. (2020) Discovery of Potent and Selective MTH1 Inhibitors for Oncology: Enabling Rapid Target (In)Validation, *ACS Medicinal Chemistry Letters.* **11**, 358-364.

12. Peng, C., Li, Y.-H., Yu, C.-W., Cheng, Z.-H., Liu, J.-R., Hsu, J.-L., Hsin, L.-W., Huang, C.-T., Juan, H.-F., Chern, J.-W. & Cheng, Y.-S. (2021) Inhibitor development of MTH1 via high-throughput screening with fragment based library and MTH1 substrate binding cavity, *Bioorganic Chemistry.* **110**, 104813.

13. Veits, G. K., Henderson, C. S., Vogelaar, A., Eron, S. J., Lee, L., Hart, A., Deibler, R. W., Baddour, J., Elam, W. A., Agafonov, R. V., Freda, J., Chaturvedi, P., Ladd, B., Carlson, M. W., Vora, H. U., Scott, T. G., Tieu, T., Jain, A., Chen, C.-L., Kibbler, E. S., Pop, M. S., He, M., Kern, G., Maple, H. J., Marsh, G. P., Norley, M. C., Oakes, C. S., Henderson, J. A., Sowa, M. E., Phillips, A. J., Proia, D. A., Park, E. S., Patel, J. S., Fisher, S. L., Nasveschuk, C. G. & Zeid, R. (2021) Development of an AchillesTAG degradation system and its application to control CAR-T activity, *Current Research in Chemical Biology.* **1**, 100010.

14. Han, Q., Campbell, R. L., Gangloff, A., Huang, Y.-W. & Lin, S.-X. (2000) Dehydroepiandrosterone and Dihydrotestosterone Recognition by Human Estrogenic 17β-Hydroxysteroid Dehydrogenase: C-18/C-19 STEROID DISCRIMINATION AND ENZYME-INDUCED STRAIN *, *Journal of Biological Chemistry.* **275**, 1105-1111.

15. Grishkovskaya, I., Avvakumov, G. V., Sklenar, G., Dales, D., Hammond, G. L. & Muller, Y. A. (2000) Crystal structure of human sex hormone‐binding globulin: steroid transport by a laminin G‐like domain, *The EMBO Journal.* **19**, 504-512.

16. Avvakumov, G. V., Muller, Y. A. & Hammond, G. L. (2000) Steroid-binding Specificity of Human Sex Hormone-binding Globulin Is Influenced by Occupancy of a Zinc-binding Site *, *Journal of Biological Chemistry.* **275**, 25920-25925.

17. Grishkovskaya, I., Avvakumov, G. V., Hammond, G. L. & Muller, Y. A. (2002) Resolution of a Disordered Region at the Entrance of the Human Sex Hormone-binding Globulin Steroid-binding Site, *Journal of Molecular Biology.* **318**, 621-626.

18. Estébanez-Perpiñá, E., Moore, J. M. R., Mar, E., Delgado-Rodrigues, E., Nguyen, P., Baxter, J. D., Buehrer, B. M., Webb, P., Fletterick, R. J. & Guy, R. K. (2005) The Molecular Mechanisms of Coactivator Utilization in Ligand-dependent Transactivation by the Androgen Receptor *<sup></sup>, *Journal of Biological Chemistry.* **280**, 8060-8068.

19. Pereira de Jésus-Tran, K., Côté, P.-L., Cantin, L., Blanchet, J., Labrie, F. & Breton, R. (2006) Comparison of crystal structures of human androgen receptor ligand-binding domain complexed with various agonists reveals molecular determinants responsible for binding affinity, *Protein Science.* **15**, 987-999.

20. Estébanez-Perpiñá, E., Arnold, L. A., Nguyen, P., Rodrigues, E. D., Mar, E., Bateman, R., Pallai, P., Shokat, K. M., Baxter, J. D., Guy, R. K., Webb, P. & Fletterick, R. J. (2007) A surface on the androgen receptor that allosterically regulates coactivator binding, *Proceedings of the National Academy of Sciences.* **104**, 16074-16079.

21. Jouravel, N., Sablin, E., Arnold, L. A., Guy, R. K. & Fletterick, R. J. (2007) Interaction between the androgen receptor and a segment of its corepressor SHP, *Acta Crystallographica Section D.* **63**, 1198-1200.

22. Zhou, X. E., Suino-Powell, K. M., Li, J., He, Y., MacKeigan, J. P., Melcher, K., Yong, E.-L. & Xu, H. E. (2010) Identification of SRC3/AIB1 as a Preferred Coactivator for Hormone-activated Androgen Receptor *<sup></sup><sup>♦</sup>, *Journal of Biological Chemistry.* **285**, 9161-9171.

23. Aka, J. A., Mazumdar, M., Chen, C.-Q., Poirier, D. & Lin, S.-X. (2010) 17β-Hydroxysteroid Dehydrogenase Type 1 Stimulates Breast Cancer by Dihydrotestosterone Inactivation in Addition to Estradiol Production, *Molecular Endocrinology.* **24**, 832-845.

24. Hsu, C.-L., Liu, J.-S., Wu, P.-L., Guan, H.-H., Chen, Y.-L., Lin, A.-C., Ting, H.-J., Pang, S.-T., Yeh, S.-D., Ma, W.-L., Chen, C.-J., Wu, W.-G. & Chang, C. (2014) Identification of a new androgen receptor (AR) co-regulator BUD31 and related peptides to suppress wild-type and mutated AR-mediated prostate cancer growth via peptide screening and X-ray structure analysis, *Molecular Oncology.* **8**, 1575-1587.

25. Nadal, M., Prekovic, S., Gallastegui, N., Helsen, C., Abella, M., Zielinska, K., Gay, M., Vilaseca, M., Taulès, M., Houtsmuller, A. B., van Royen, M. E., Claessens, F., Fuentes-Prior, P. & Estébanez-Perpiñá, E. (2017) Structure of the homodimeric androgen receptor ligand-binding domain, *Nature Communications.* **8**, 14388.

26. Alegre-Martí, A., Jiménez-Panizo, A., Martínez-Tébar, A., Poulard, C., Peralta-Moreno, M. N., Abella, M., Antón, R., Chiñas, M., Eckhard, U., Piulats, J. M., Rojas, A. M., Fernández-Recio, J., Rubio-Martínez, J., Le Romancer, M., Aytes, Á., Fuentes-Prior, P. & Estébanez-Perpiñá, E. (2023) A hotspot for posttranslational modifications on the androgen receptor dimer interface drives pathology and anti-androgen resistance, *Science Advances.* **9**, eade2175.

27. Doamekpor, S. K., Peng, P., Xu, R., Ma, L., Tong, Y. & Tong, L. (2023) A partially open conformation of an androgen receptor ligand-binding domain with drug-resistance mutations, *Acta Crystallographica Section F.* **79**, 95-104.

28. Svensson, L. M., Jemth, A.-S., Desroses, M., Loseva, O., Helleday, T., Högbom, M. & Stenmark, P. (2011) Crystal structure of human MTH1 and the 8‐oxo‐dGMP product complex, *FEBS letters.* **585**, 2617-2621.

29. Waz, S., Nakamura, T., Hirata, K., Koga-Ogawa, Y., Chirifu, M., Arimori, T., Tamada, T., Ikemizu, S., Nakabeppu, Y. & Yamagata, Y. (2017) Structural and Kinetic Studies of the Human Nudix Hydrolase MTH1 Reveal the Mechanism for Its Broad Substrate Specificity, *Journal of Biological Chemistry.* **292**, 2785-2794.

30. Jemth, A. S., Gustafsson, R., Brautigam, L., Henriksson, L., Vallin, K. S. A., Sarno, A., Almlof, I., Homan, E., Rasti, A., Warpman Berglund, U., Stenmark, P. & Helleday, T. (2018) MutT homologue 1 (MTH1) catalyzes the hydrolysis of mutagenic O6-methyl-dGTP, *Nucleic Acids Res.* **46**, 10888-10904.

31. Nakamura, T., Hirata, K., Fujimiya, K., Chirifu, M., Arimori, T., Tamada, T., Ikemizu, S. & Yamagata, Y. (2019) X-ray Structure Analysis of Human Oxidized Nucleotide Hydrolase MTH1 using Crystals Obtained under Microgravity, *International Journal of Microgravity Science and Application.* **36**, 360103.

32. Scaletti, E. R., Vallin, K. S., Brautigam, L., Sarno, A., Warpman Berglund, U., Helleday, T., Stenmark, P. & Jemth, A. S. (2020) MutT homologue 1 (MTH1) removes N6-methyl-dATP from the dNTP pool, *J Biol Chem.* **295**, 4761-4772.

33. Nakamura, T., Koga-Ogawa, Y., Fujimiya, K., Chirifu, M., Goto, M., Ikemizu, S., Nakabeppu, Y. & Yamagata, Y. (2023) Protonation states of Asp residues in the human Nudix hydrolase MTH1 contribute to its broad substrate recognition, *FEBS Letters.* **597**, 1770-1778.
